# The potential biomarkers of organ transplantation in the era of machine perfusion: Current status, challenges, and future directions

**DOI:** 10.1016/j.isci.2026.117835

**Published:** 2026-10-28

**Authors:** Zongwan Yang, Xiaoqing Ying, Zhaojie Guan, Shangxin Dong, Yuanyuan Zhao, Huibo Shi, Jipin Jiang

**Affiliations:** 1 Institute of Organ Transplantation, Tongji Hospital, Tongji Medical College, Huazhong University of Science and Technology, Key Laboratory of Organ Transplantation of Ministry of Education, National Health Commission and Chinese Academy of Medical Sciences, Wuhan, China; 2 Organ Transplantation Center, Shanxi Bethune Hospital, Taiyuan, China; 3 Department of Organ Donation, Tongji Hospital, Tongji Medical College, Huazhong University of Science and Technology, Key Laboratory of Organ Transplantation, Ministry of Education, NHC Key Laboratory of Organ Transplantation, Key Laboratory of Organ Transplantation, Chinese Academy of Medical Sciences, Organ Transplantation Clinical Medical Research Center of Hubei Province, Wuhan, China

**Keywords:** biomarker, machine perfusion, organ transplantation, viability assessment

## Abstract

Machine perfusion (MP) is a technology that mimics the physiological state in an *ex vivo* environment to preserve organs and enable their dynamic assessment. Biomarkers measured during MP hold significant promise for predicting post-transplant outcomes and guiding decisions on organ utilization. However, the clinical translation of this field is currently hindered by a lack of robust research defining the relevant biomarkers, their types, and their clinically applicable thresholds. This review outlines the development and fundamental principles of MP devices and provides a definition and classification of biomarkers. We systematically summarize and categorize potential MP biomarkers for the liver, kidney, lung, heart, and pancreas. Current research into MP biomarkers remains insufficient, and its clinical application is constrained by factors including MP platform heterogeneity and donor variability. Further studies are needed to determine the sensitivity, specificity, stability, and reproducibility, while exploring dynamic monitoring for timely assessment and intervention during MP.

## Graphical abstract

**Figure:**
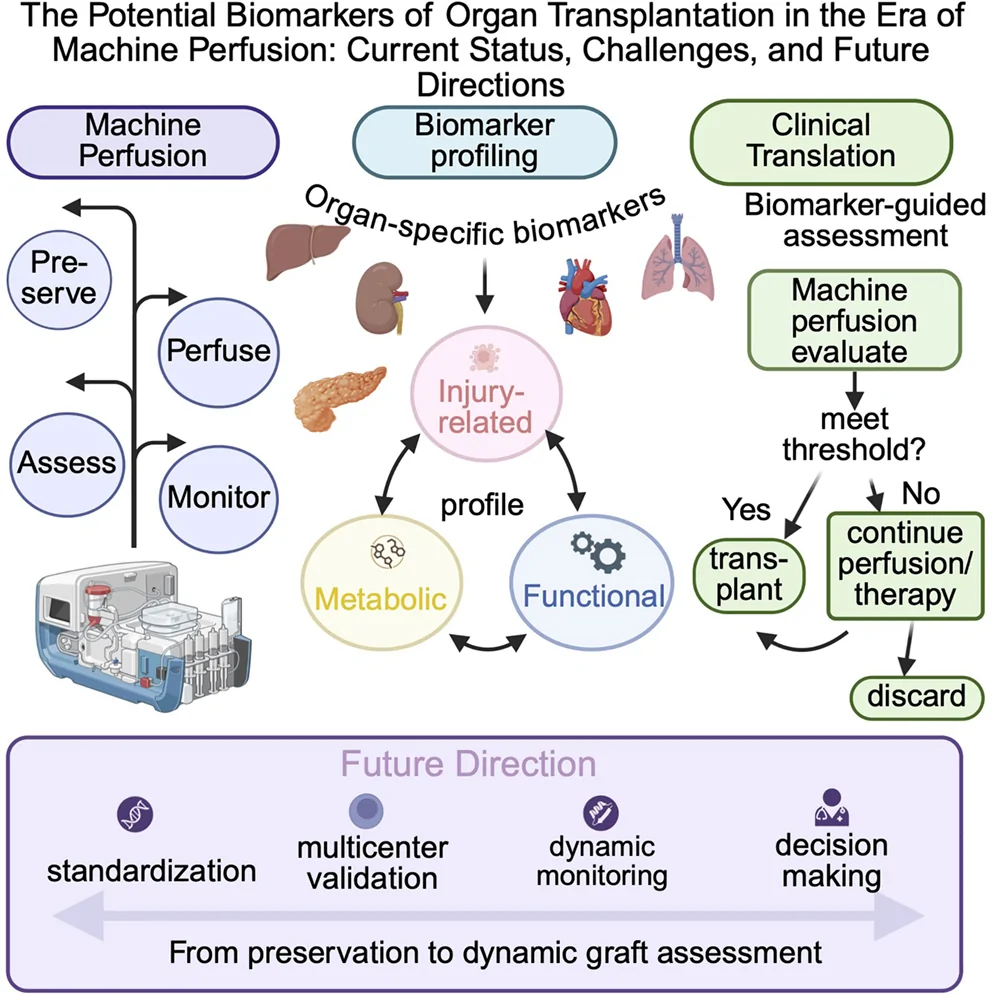

## Teaser

Health sciences; Medicine; Medical specialty; Surgery

## Introduction

Transplantation is severely limited by the shortage of donor grafts. Although donation after brain death (DBD) provides optimal grafts, the number of such donors cannot meet the growing demand from patients on waiting lists. The use of extended criteria donors (ECDs) has expanded the donor pool, and many studies have demonstrated favorable outcomes with these grafts.^1^ Nevertheless, ECD livers are associated with a higher risk of early allograft dysfunction (EAD) and primary non-function due to ischemia-reperfusion injury (IRI).^2^ Machine perfusion (MP) represents a novel approach to organ preservation in transplantation. Based on perfusion temperature, MP is categorized into normothermic MP (NMP), hypothermic MP (HMP), and subnormothermic MP (SNMP), with temperatures maintained at 35°C–37°C, 4°C–12°C, and 20°C–25°C, respectively.^3^^,^^4^^,^^5^^,^^6^ Among these, NMP has been shown to mitigate ischemic injury and its associated complications.^7^ Consequently, NMP is increasingly being used for the preservation and evaluation of livers,^8^ kidneys,^9^ lungs,^10^ and heart^11^ in clinical research.

MP equipment not only serves as a platform for organ transport and preservation but, through its continuous perfusion and real-time monitoring capabilities, also enables the dynamic collection of biomarkers. These biomarkers, including diagnostic, monitoring, pharmacodynamic, predictive, safety, and susceptibility types, hold tremendous clinical value.^12^ Consequently, the study of biomarkers in MP is of great importance for assessing organ quality and predicting post-transplant prognosis. In renal transplantation (RT), the estimated glomerular filtration rate (eGFR) measured during NMP has been shown to correlate strongly with post-transplant outcomes.^13^ Similarly, several studies have highlighted the potential of biomarkers to predict early graft function in liver transplantation (LT).^14^^,^^15^ However, biomarker-guided modulation of MP protocol remains largely unexplored.

Existing reviews have primarily summarized biomarker types, paying little attention to their clinical translation. We summarize biomarkers identified during MP across different organs. In addition, we compare biomarker performance across organs and assess their translational potential. Finally, we outline the translational approaches and prospects for integrating biomarkers into organ quality assessment.

## The development history of MP

### The definition of MP

MP is a technique that mimics physiological conditions *in vitro*. Compared with static cold storage (SCS), MP provides an environment that better approximates normal physiological conditions prior to transplantation. Within the MP circuit, a pump emulates cardiac output by propelling perfusate through the organ’s arterial supply. The perfusate may pass through an oxygenator and a temperature regulation unit to control oxygen content and temperature before entering the organ.

### Development history of MP

The development of MP has been both prolonged and complex (Figure 1). In the history of organ preservation research, SCS has long dominated clinical practice. However, with the advent of specialized perfusion solutions, improvements in manufacturing processes, and technological advances, MP has demonstrated considerable potential for organ protection and quality assessment. As early as the 1850s, Claude Bernard described equipment for liver perfusion.^16^ Then a major milestone came with the Lindbergh perfusion pump, which enabled sterile organ perfusion and marked the beginning of a new era in MP technology.^17^ Building on these advances, the first successful *ex situ* MP experiments were achieved in the liver in 1966^18^ and in the kidney in 1968.^19^ Until now, long-term perfusion, spanning days to weeks, provides a window for organ modification prior to transplantation.^20^^,^^21^^,^^22^ Long-term NMP provides physicians with a time window for assessing organ function, but traditional parameters such as flow rate, temperature, and pressure do not provide a comprehensive assessment of organ condition. Consequently, the introduction of objective, quantitative biomarkers has become key to the *in vitro* assessment of organ quality.

**Figure 1:**
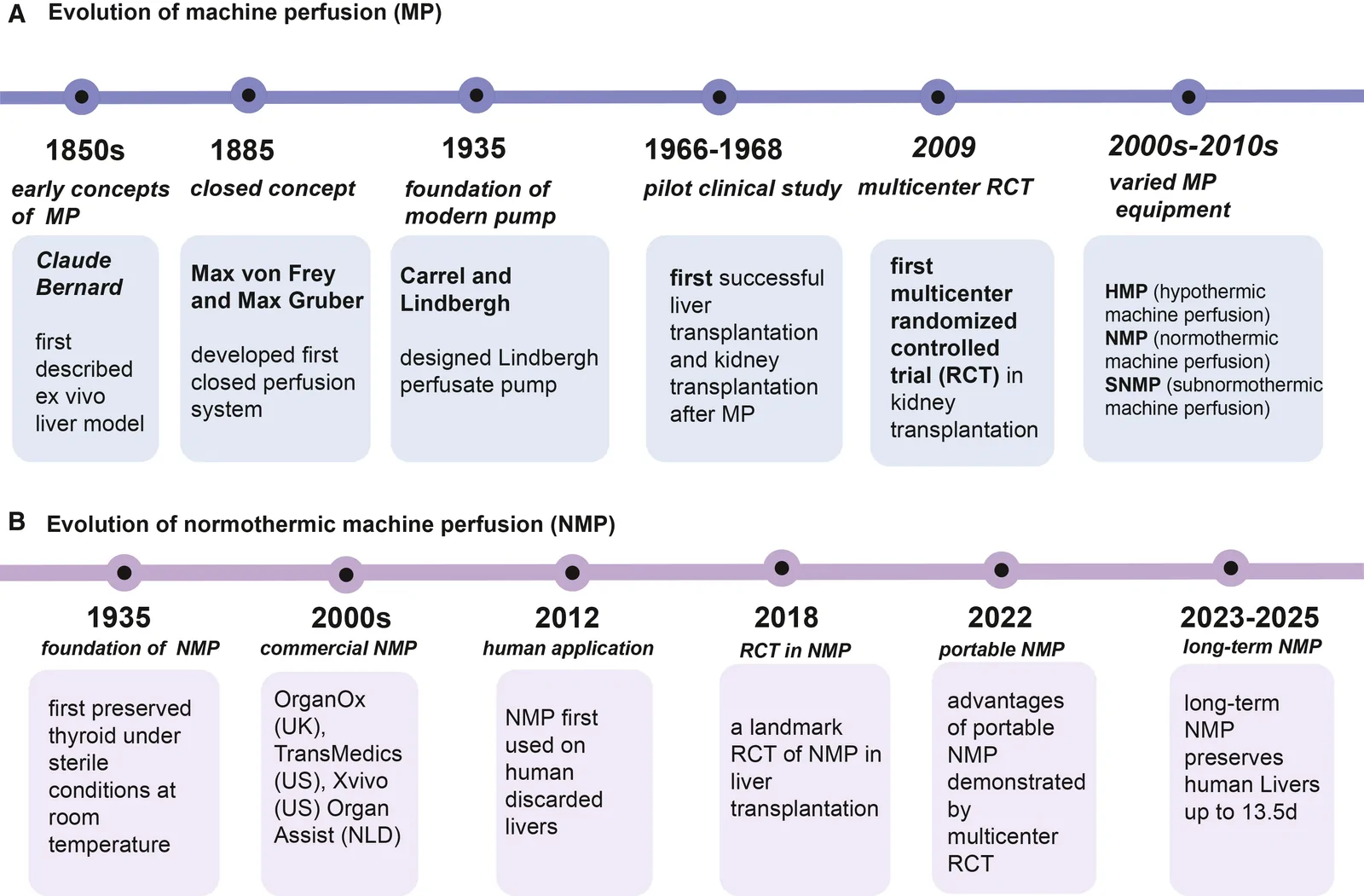
Historical evolution of machine perfusion and normothermic machine perfusion (A) Major milestones in the evolution of machine perfusion (MP). (B) Timeline of key advances of normothermic machine perfusion (NMP), from foundational concepts to clinical application. For a complete list of references incorporated in this figure, see the supplemental information.

A conventional MP system (Figure 2) typically comprises a sterile organ chamber, one or more pumps, a temperature controller, an oxygenator, and sensors for real-time monitoring of perfusion parameters.^23^^,^^24^^,^^25^ Some studies have incorporated additional components, such as dialysis cassettes, arterial filters, and reservoirs.^26^^,^^27^

**Figure 2:**
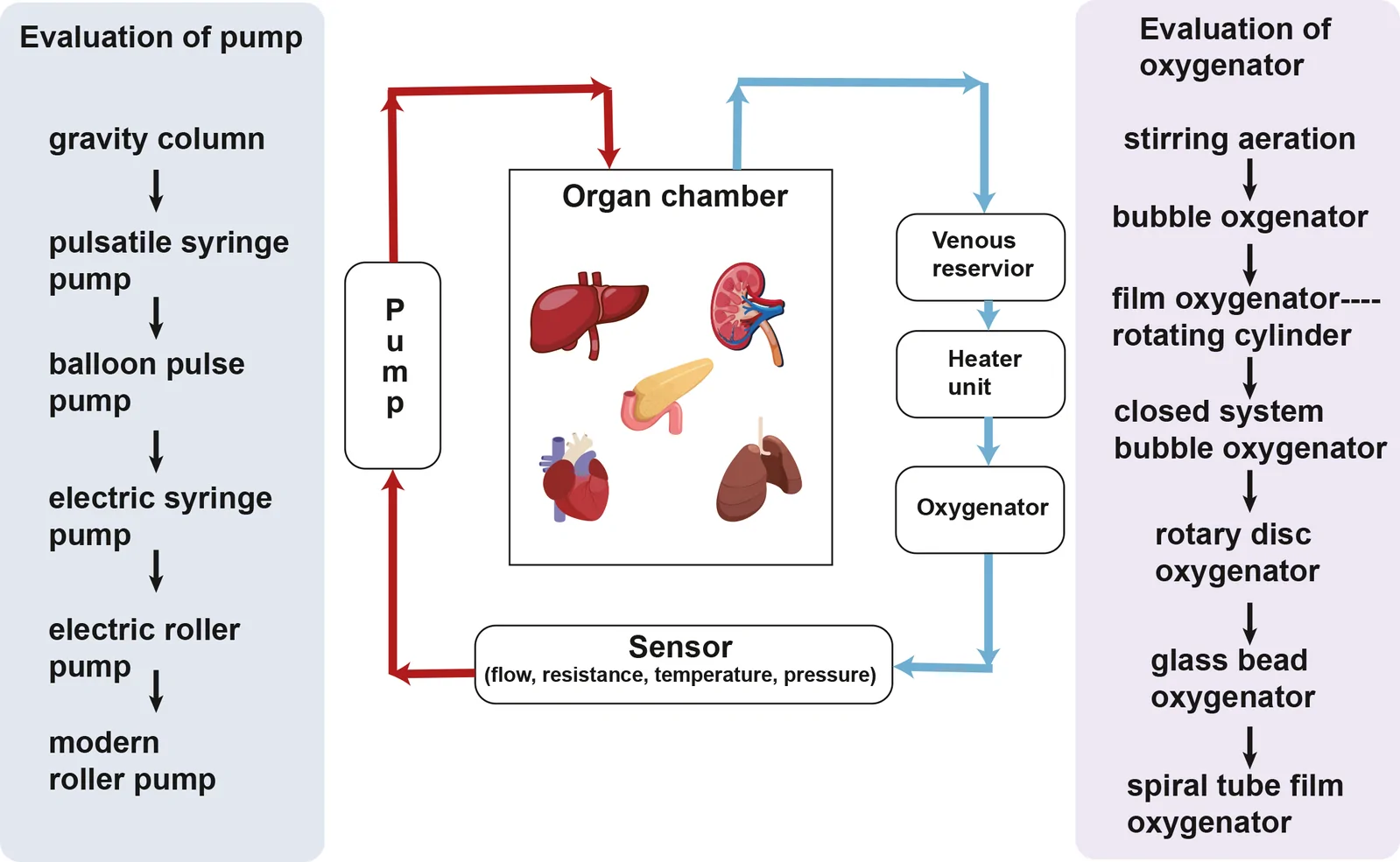
Schematic illustration of MP and its historical evolution The central schematic illustrates the perfusion circuit of machine perfusion system (MP). MP simulates physiological blood flow by pumping perfusate into an organ housed in a sterile chamber. The effluent is collected after passing through the organ and undergoes temperature regulation and oxygenation before recirculation. Sensors continuously monitor flow rate, vascular resistance, temperature, and perfusion pressure throughout the process. The side images illustrate the technological evolution of perfusion pumps (left), from gravity-driven and pulsatile systems to modern roller pumps, and oxygenation devices (right), from early aeration and bubble-based approaches to more advanced oxygenator designs.

### Paradigms in the evolution of MP and unmet needs

MP has evolved from a preservation technology into a platform for dynamic organ recovery and intervention.

The goal of MP is to expand the donor pool, particularly by facilitating the use of ECD and donation after circulatory death (DCD) organs, which demand accurate assessment. There is an urgent need to establish a standardized, objective, and quantitative biomarker system for assessing organ viability during MP. Traditional MP parameters, such as perfusion flow rate, vascular resistance, and perfusion temperature, are already used as monitoring indicators. However, current biomarkers lack the specificity, sensitivity, and validated predictive thresholds required for reliable, personalized clinical decision-making. Thus, the identification, validation, and clinical integration of robust biomarkers could transform MP from a passive preservation method into an active decision-support system.

## Biomarkers in MP

### Biomarkers in MP: Concept, classification, and core value

Biomarkers are generally defined as objectively measurable indicators that reflect normal physiological processes, pathological alterations, or pharmacological responses to therapeutic interventions.^28^

A qualified biomarker must meet several criteria: specificity, sensitivity, stability, repeatability, and clinical utility.^29^^,^^30^^,^^31^ Biomarkers may serve different clinical roles depending on the context of their application (Figure 3).

**Figure 3:**
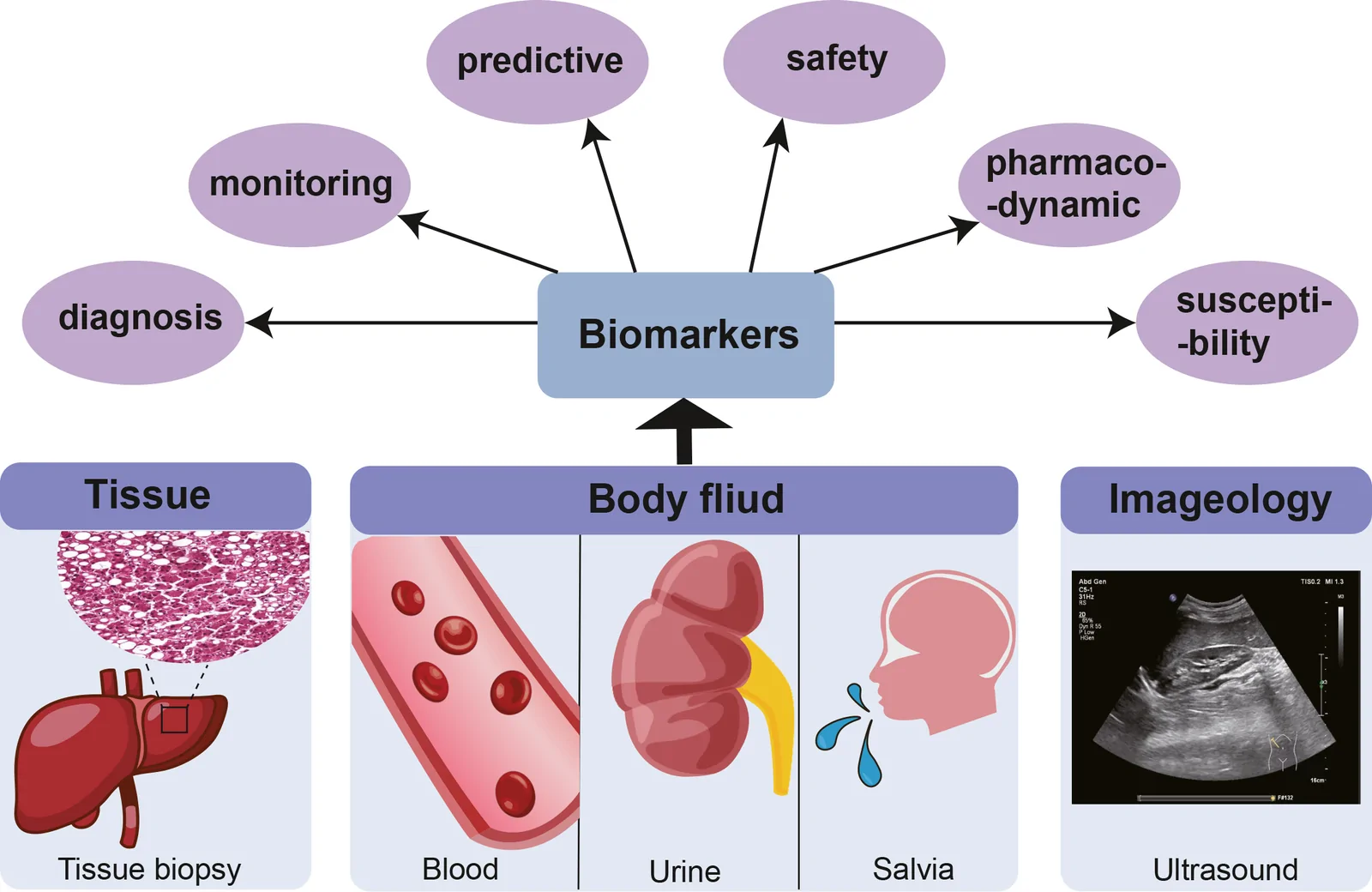
Classification of biomarkers by application and their primary clinical sources In spring 2015, the FDA-NIH Joint Leadership Council addressed classification of biomarkers by developing the BEST (biomarkers, endpoints, and other tools) Resource, which provides a structured classification system and clarifies distinctions between biomarkers and clinical endpoints. Biomarkers are classified into 7 categories including diagnostic, monitoring, prognostic, predictive, safety, pharmacodynamic, and susceptibility. In clinical practice, biomarkers are derived from a variety of source, including tissue, imaging modalities, blood, urine, and saliva.

MP serves as an independent, *in vitro* organ preservation system; the perfusion fluid, urine, bile, and other fluids all originate from a single organ and are not affected by other organs or the body’s internal environment. The biomarkers used to reflect the condition of different organs vary, but overall, assessment is carried out in terms of three aspects: damage, metabolism, and function.

### Biomarkers of different organs in MP

#### Liver

In LT, biomarkers such as transaminases, bilirubin, platelet count, and international normalized ratio (INR) are used to evaluate clinical outcomes and are incorporated into prognostic scoring systems including EAD,^32^ the Model for Early Allograft Function (MEAF),^33^ Liver Graft Assessment Following Transplantation (L-GrAFT),^34^ and Early Allograft Failure Simplified Estimation (EASE).^35^

In clinical research, the main biomarkers used to determine whether a liver after MP is suitable for transplantation are lactate, aspartate aminotransferase (AST), alanine aminotransferase (ALT), and lactate dehydrogenase (LDH).^32^^,^^36^

##### Lactate as a functional metabolic biomarker

Lactate metabolism plays a key role in evaluating liver quality, reflecting hepatocellular functions such as gluconeogenesis and mitochondrial oxidative capacity. During MP, livers are exposed to both warm and cold ischemia, which disrupts lactate homeostasis. Under hypoxic conditions *ex vivo*, the absence of circulation leads to lactate accumulation. Upon reperfusion, livers with intact mitochondrial function rapidly metabolize accumulated lactate through aerobic oxidation once oxygen and energy substrates are restored. Thus, both lactate levels and clearance kinetics serve as indicators of hepatocellular metabolic function.

Clinically, studies using MP have established lactate thresholds for assessing organ quality (Table 1). In NMP, previous studies set strict criteria: lactate <2.5 mmol/L within 2 h.^41^^,^^42^ The landmark VITTAL trial widely adopted a threshold of lactate clearance to ≤2.5 mmol/L within 4 h to increase the utilization of discarded livers.^37^ However, that trial also monitored additional parameters such as perfusate pH and glucose levels. Other studies reported alternative thresholds, for example, >60% lactate reduction and absolute values < 4.0 mmol/L within 3 h during continuous hypothermic oxygenated machine perfusion (HOPE)-NMP.^38^ In a single-center study, livers were discarded if lactate exceeded 4.1 mmol/L within 4 h of NMP.^39^ For severely steatosis livers, the observation period for stable lactate clearance must be extended to 6 h.^43^ These findings confirm that lactate thresholds are not universally applicable across different contexts.

**Table 1:** Perfusate lactate threshold in liver transplantation

Study	Research subjects	MP type	Assessment point	Threshold	Clinical outcomes
VITTAL clinical trials^37^; Single center	discard livers	NMP	within 4 h	lactate clearance ≤2.5 mmol/L	viability of transplantation; 90 days survival
Liu et al.^38^ Single center	ECD DCD	continuous HOPE-NMP	within 3 h in NMP	lactate clearance< 60% and level <4.0 mmol/L	viability of transplantation; EAD; graft failure or patient death; ischemic cholangiopathy
Quintini et al.^39^ Single center	discard livers	NMP	after 3 h in NMP	lactate <4.1 mmol/L	viability of transplantation; early postoperative complications; PNF; EAD; ischemic cholangiopathy
Hofmann et al.^40^ Multicenter study	DBD DCD	NMP	at 6 and 0–6 h	lactate levels and AUC significantly correlated with MEAF score	predictive efficacy of lactate clearance on graft function

MP, machine perfusion; NMP, normothermic machine perfusion; ECD, extended criteria donor; DCD, donation after circulatory death; HOPE, hypothermic oxygenated machine perfusion; EAD, early allograft dysfunction; PNF, primary nonfunction; DBD, donation after brain death; AUC, area under the curve; MEAF, model for early allograft function.

Recent studies emphasize dynamic monitoring of lactate. One study found that lactate levels at 6 h and the area under the curve (AUC) from 0 to 6 h correlated strongly with the MEAF score (r = 0.3176, *p* < 0.0001), with predictive performance improving as perfusion duration increased.^40^ Dynamic assessment, rather than reliance on a single time point, enables earlier detection of decompensated livers, thereby reducing the risk of primary nonfunction (PNF).

Variability in lactate thresholds stems from multiple factors: heterogeneity in organ quality, differences in MP devices, outcome definitions, perfusion duration, and institutional tolerance for clinical risk. For instance, the VITTAL trial’s use of discarded livers led to stricter lactate criteria,^37^ whereas Liu et al. applied more lenient thresholds for ECD livers.^38^ In fatty livers, hepatocellular metabolic impairment renders single-time point lactate measurements less reliable than dynamic clearance patterns. Additionally, MP parameters, including device type, perfusion pressure, flow rate, oxygenation method, temperature, perfusate composition, and baseline lactate levels, all influence lactate metabolism and thus affect threshold interpretation. Outcome measures also contribute to threshold variation. MEAF is a continuous variable, whereas EAD and PNF are binary endpoints; the choice of statistical approach may therefore yield different optimal cutoffs. For example, Hofmann et al. reported that 6-h lactate levels outperformed 2-h values in predicting MEAF (r = 0.3176 vs. 0.1734).^40^ However, the VITTAL trial limited MP to 4 h, making its lactate threshold incomparable to studies with longer perfusion times. Clinical risk tolerance further shapes threshold selection. The Cambridge and Groningen protocols achieved high specificity but low sensitivity (44% and 56%, respectively), potentially leading to over-discarding of viable grafts. In contrast, the Birmingham protocol showed 94% sensitivity but only 50% specificity, suggesting that some nonviable livers may have been transplanted.^44^ Such differences in risk acceptance directly influence lactate threshold adoption.

As a potential MP biomarker, lactate offers advantages including real-time monitoring capability, low cost, and strong predictive value. Nevertheless, its limitations must be acknowledged. Lactate does not reflect cholangiocyte function; ischemic biliary complications can still occur despite adequate lactate clearance before LT.^37^ Moreover, lactate is more indicative of cellular injury than of early functional recovery.^45^ In HOPE, lactate levels showed poor performance in predicting EAD and transaminase elevation^46^ and should not be used as a sole decision-making criterion.^47^

As a biomarker that can be measured in real time in a clinical setting, lactate is now widely used in liver MP and has a solid foundation for clinical application. However, lactate cannot be used as a standalone decision-making indicator, as its predictive value is limited. Liver quality should be assessed by considering a combination of factors, including lactate levels, bile parameters, transaminases, and perfusion kinetics. Future research should focus not only on determining thresholds but also on establishing standardized testing procedures and uniform assessment criteria and integrating these into predictive models.

##### Mitochondrial injury-related biomarkers

Mitochondria play a central role in cellular energy metabolism and IRI; thus, mitochondrial function reflects graft viability. In liver MP, flavin mononucleotide (FMN) has emerged as a promising biomarker due to its ability to specifically indicate damage to complex I of the mitochondrial respiratory chain. During MP, FMN is released from injured mitochondria into the perfusate. Impaired liver function is characterized by low intracellular FMN levels and elevated perfusate concentrations.^48^ In a preclinical study using HOPE-treated livers from DCD mice, perfusate FMN levels were lower than those observed under NMP, demonstrating that HOPE mitigates mitochondrial injury.^49^

In clinical studies, a single-center trial reported strong predictive performance of perfusate FMN for severe graft dysfunction (AUC: 0.79) and early graft loss (AUC: 0.93). Its accuracy surpassed that of the traditional Donor Risk Index (DRI) and L-GrAFT score, with a proposed rejection threshold of 10,000 a.u. (equivalent to 100 ng FMN/mL).^50^ Subsequently, a multicenter study showed that FMN measured at 60 min during MP predicted graft loss (c-statistic 0.84) and outperformed both DRI and the Balance of Risk (BAR) score in predicting symptomatic anastomotic stricture and post-transplant renal failure requiring renal replacement therapy (AUC = 0.75 and 0.76, respectively). This study also defined three risk categories based on perfusate FMN: low risk (<5,165 a.u.), intermediate risk, and high risk (>6,415 a.u.).^48^ A prospective cohort study found that FMN levels measured just 5 min after initiating HOPE effectively predicted EAD (AUC: 0.744) and 1-year patient survival (AUC: 0.890). In multivariate regression analysis, perfusate FMN was the sole independent predictor of 1-year survival (odds ratio [OR] = 1.115, *p* = 0.018). The study established three risk thresholds for EAD and 1-year survival: low risk (<10.6 ng/mL; EAD incidence 8%, 1-year survival 100%), intermediate risk (10.6–23.5 ng/mL; EAD incidence 47%, 1-year survival 100%), and high risk (>23.5 ng/mL; EAD incidence 62%, 1-year survival 38%).^15^ These findings highlight the potential of perfusate FMN to support early, high-accuracy clinical decision-making. In another single-center prospective study, perfusate FMN was confirmed as a predictor of graft failure (AUC = 0.96), with a high-risk threshold for post-transplant survival defined as >1.75 μg/mL at 4 h.^51^

Although current studies have established predictive thresholds for perfusate FMN, these values vary across reports (Table 2). This variability is linked to donor liver quality and baseline perfusate FMN levels. For instance, in the multicenter study, the Zurich center reported higher initial FMN levels in discarded livers compared with other sites. Additionally, median perfusate FMN was higher in DCD than in DBD grafts.^48^ Differences in pre-MP interventions or MP protocols may also contribute to threshold variation. For example, when donor livers undergo normothermic regional perfusion (NRP) prior to HOPE, the use of blood-containing perfusate introduces free hemoglobin, which interferes with fluorometric FMN measurement and increases false-positive readings.^48^ Detection methodology further influences results. Perfusate FMN can be quantified by fluorimetry or liquid chromatography-mass spectrometry (LC-MS), which differ in detection principles, numerical outputs, and units, leading to non-comparable threshold values. To address this, Sun et al.^52^ proposed a standardized spectrophotometric protocol for FMN measurement in donor plasma and perfusate, detailing sample preparation, interference management, and quality control measures to enhance cross-study comparability and reproducibility. Moreover, FMN sampling time points differ across studies. While early measurements show promise, the dynamic trajectory of perfusate FMN requires further investigation to validate its predictive value over time.

**Table 2:** Perfusate FMN threshold in liver transplantation

Study	Research subjects	MP type	Assessment point	Threshold	Clinical outcomes
Muller et al.^50^ Single center	DCD DBD	HOPE	30 min	10,000 a.u. (100 ng FMN/mL)	predictive efficacy of graft function
Eden et al.^48^ Multicenter study	DCD DBD	HOPE	60 min	low risk, < 5,165 a.u.; medium and high risk, >6,415 a.u.	predictive efficacy of graft loss
Dingfelder et al.^15^ Single center	DBD	HOPE	5 min	low risk, <10.6 ng/mL; intermediate risk, 10.6–23.5 ng/mL; high risk, >23.5 ng/mL	predictive efficacy of EAD and 1-year mortality
Wehrle et al.^51^ Single center	all liver transplants	NMP	4 h	high risk >1.75 μg/mL	predictive efficacy of graft loss

FMN, flavin mononucleotide; MP, machine perfusion; DCD, donation after circulatory death; DBD, donation after brain death; HOPE, hypothermic oxygenated machine perfusion; EAD, early allograft dysfunction; NMP, normothermic machine perfusion.

Future clinical applications of perfusate FMN should be based on standardized MP protocols and consistent detection methods. Prospective studies remain limited, particularly regarding the dynamic changes in FMN during perfusion. Although spectrophotometry can detect FMN very quickly, it does not allow for bedside testing. Combining perfusate FMN with other biomarkers may enable a more comprehensive assessment of liver graft quality.

##### Biliary biomarkers

Biliary complications are among the most common postoperative complications after LT, adversely affecting patients’ quality of life and healthcare costs and potentially leading to graft loss. The bile ducts are highly susceptible to IRI. However, conventional biomarkers of cellular damage, such as transaminases and bilirubin, lack specificity for biliary function. Consequently, the development of bile-duct-specific biomarkers during MP holds considerable research potential.

Current biliary biomarkers in MP primarily include bile bicarbonate concentration, pH, and glucose levels, reflecting the physiological functions of cholangiocytes to secrete bicarbonate (thereby alkalinizing bile) and reabsorb glucose. In a preclinical study of 23 human livers, correlation analyses were performed between these bile parameters and histological markers of bile duct injury. The study confirmed that bile bicarbonate, pH, and glucose levels correlated with bile duct damage and established specific thresholds at 2 h of NMP: bile bicarbonate >18 mmol/L, pH > 7.48, glucose <16 mmol/L, or a bile-to-perfusate glucose ratio <0.67. These criteria were prospectively applied to six livers; four that met the thresholds remained free of biliary complications postoperatively.^53^ Although this study included clinical validation, its sample size was small, and external validation is needed. Notably, while bile biomarkers showed strong correlation with the biliary duct injury (BDI) score, no direct association with postoperative biliary complications was demonstrated. Reported thresholds for bile pH also vary. In a prospective clinical trial involving 16 discarded human livers preserved with DHOPE-COR-NMP, a bile pH > 7.45 was used as one of the transplant eligibility criteria.^54^ This discrepancy appears related to graft quality. In another study by de Jong et al.,^55^ which evaluated 42 discarded livers treated with DHOPE-COR-NMP, absolute bile measurements were not used. Instead, differences between bile and perfusate levels were assessed, with the following thresholds proposed for transplantation: ΔpH >0.10, ΔHCO_3_^−^ > 5.0 mmol/L, and Δglucose <−5.0 mmol/L. That study also found that transplantable livers exhibited better preservation of peribiliary glands (PBGs), higher cholangiocyte proliferation (Ki-67+), and a more intact peribiliary microvascular network. Beyond traditional markers, a novel approach using biliary cytokines has been proposed to assess bile duct regeneration. One study reported that the maintenance, not the absolute concentration, of IL-6 and VEGF-A levels in bile was significantly associated with bile duct regeneration (*p* = 0.017).^22^ However, the sample size was limited, and long-term post-LT follow-up was lacking, underscoring the need for further prospective validation.

Additionally, bile glutathione (GSH) levels are inversely correlated with the incidence of biliary complications. Transplantable livers showed a significant increase in bile GSH during NMP (from 25.9 μM at 1 h to 108.7 μM at endpoint, *p* < 0.001), whereas discarded livers did not demonstrate this upward trend. Moreover, bile GSH levels below 78.4 μM at 6 h of NMP were significantly associated with a higher risk of biliary complications within 1 year post-transplantation (*p* = 0.022).^56^ These findings suggest that dynamic changes in bile GSH not only reflect cholangiocyte activity but also serve as a quantitative indicator of their resistance to oxidative stress, though this requires confirmation in future multicenter prospective studies.

Rapid point-of-care testing devices for bile pH and bile glucose are already available, and many centers are able to carry out these tests immediately, giving them potential clinical translational value. To date, research on biliary biomarkers in MP has been limited to single-center, small-scale studies. Biomarker utility is constrained by bile production volume, and thresholds for traditional biomarkers remain inconsistent. Therefore, multicenter, prospective randomized trials are warranted to establish standardized biliary biomarkers for clinical use. The aim of clinical translation lies in establishing the process by which clinicians assess organ quality, from the collection of bile samples, through the determination of testing methods and timing, to the establishment of assessment thresholds.

##### Molecular and immune biomarkers

Cell-free DNA (cfDNA) has shown predictive value for assessing IRI in MP studies of LT. Westhaver et al. reported that during NMP, perfusate mitochondrial DNA (mtDNA) levels were significantly positively correlated with the severity of donor liver injury, as well as with donor INR (ρ = 0.43, *p* = 0.02), lactate (ρ = 0.41, *p* = 0.03), and warm ischemia time (ρ = 0.51, *p* = 0.004), and negatively correlated with inferior vena cava flow. The study also found that bile mtDNA concentrations were approximately 1,000-fold higher than those in perfusate, with cholangiocyte epithelium identified as the primary source.^57^ This positions bile mtDNA as a highly promising, bile-duct-specific biomarker for predicting post-transplant biliary complications and warrants further investigation.

Donor-derived cfDNA (ddcfDNA) also shows potential for evaluating liver quality. During the first 4.25 h of NMP, plasma ddcfDNA levels rose sharply in all livers due to reperfusion injury. However, during the critical decision-making window between 4.25 and 6.25 h of NMP, ddcfDNA continued to increase in transplantable livers, reaching 117.15 × 10^6^ copies/mL, whereas it declined to 16.72 × 10^6^ copies/mL in discarded livers (*p* = 0.01). These findings suggest that sustained, moderate DNA release may reflect active cellular clearance and repair processes rather than uncontrolled necrosis. Notably, bile ddcfDNA exhibited a decreasing trend in viable livers,^58^ offering new insights into biliary tract function assessment. In contrast, a D-HOPE study found that low postoperative plasma dd-cfDNA levels were associated with early adverse outcomes and complications and that perfusate mt-cfDNA was a strong predictor of poor graft outcomes.^59^ This apparent discrepancy may stem from differences in the type of injury induced by distinct MP modalities, particularly perfusion temperature. Therefore, when selecting biomarkers to guide clinical decisions, consideration should be given to variations among MP platforms and sampling time points.

Lower initial perfusate hemoglobin levels were positively associated with the incidence of EAD and elevated transaminases, both of which declined during NMP.^60^ Other blood-derived components in perfusate also show predictive potential: platelet count and von Willebrand factor (vWF) antigen were identified as indicators of EAD.^61^ Bulk RNA sequencing of perfusate samples revealed seven genes with high predictive value for graft quality, among which CD274 (encoding programmed death-ligand 1, PD-L1) demonstrated the strongest association.^14^ In addition, soluble PD-1 (sPD-1), the primary ligand of PD-1, was correlated with IRI.^62^

At present, these molecule biomarkers suffer from the drawbacks of complex detection methods, high costs, and a lack of real-time capability, and their application is therefore limited to the laboratory setting. Although the clinical translational value of these biomarkers is limited, they provide important clues and insights into the potential mechanisms underlying delayed graft function (DGF), PNF, and EAD.

#### Kidney

##### Traditional injury biomarkers

In clinical studies, perfusate levels of LDH, neutrophil gelatinase-associated lipocalin (NGAL), and glutathione S-transferase (GST) have been associated with post-transplant outcomes. In a single-center study of 43 DBD kidneys preserved with HMP, perfusate NGAL predicted DGF (AUC = 0.766, *p* = 0.012), while perfusate LDH predicted PNF (AUC = 0.84, *p* = 0.027).^63^ However, the study did not specify the timing of perfusate sampling, and variations in sampling time may have introduced bias. Qiao et al.^64^ analyzed perfusion parameters and perfusate samples collected 4 h after initiation of HMP in 113 kidney transplant recipients and identified end-stage HMP resistance (OR = 1.879) and GST (OR = 1.62) as independent risk factors for DGF. Combining these two variables yielded an AUC of 0.888 for DGF prediction, significantly outperforming either marker alone. Another single-center clinical study reported that the delta changes in serum GST-Pi correlated significantly with eGFR at 12 months post-transplantation (*R* = 0.54, *p* = 0.001), suggesting its association with long-term renal function.^13^

To date, research on traditional biomarkers has been limited to single-center, small-scale clinical studies, with a lack of prospective multicenter validation. LDH, a general marker of cellular damage, is simple, inexpensive, and routinely measured in clinical practice. However, it lacks tissue specificity and cannot localize the site of injury within the transplanted kidney. Consequently, the predictive value of LDH alone is limited when assessing the extent of kidney injury. Taking into account the available evidence, the feasibility of testing, and clinical application scenarios, although LDH is not yet sufficient to serve as an independent decision-making indicator for assessing renal graft quality, its good clinical accessibility and standardized testing infrastructure confer upon its significant translational potential. In the future, following validation through multicenter clinical cohorts and optimization of thresholds, LDH may serve as a key component of a multi-indicator combined assessment system, used to improve the accuracy of graft quality assessment during MP. Moreover, its stability in perfusate is temperature dependent, and reported threshold values vary across perfusion conditions. NGAL is an early and sensitive marker of acute kidney injury, primarily derived from renal tubular epithelial cells, and shows relative specificity for ischemic injury. Nevertheless, under inflammatory conditions, NGAL is also secreted by neutrophils and other epithelial cells, which may confound the identification of its renal origin. Although NGAL is not yet a routine clinical marker in the assessment of MP of renal grafts—due to its relatively high testing costs and the fact that testing procedures have not yet been fully standardized—its high sensitivity and specificity for early tubular damage give it considerable potential for clinical translation. Nevertheless, the further application of NGAL in clinical decision-making requires the resolution of several key issues, including the establishment of effective predictive thresholds based on MP scenarios, the clarification of its association with graft function recovery and long-term transplant outcomes, and the optimization of testing methods to improve testing efficiency and reduce application costs. Through validation in future multicenter prospective studies, NGAL is expected to serve as a key component of a combined biomarker assessment system, used to enhance the accuracy of donor kidney quality assessment during MP. GST exhibits region-specific expression: α-GST is predominantly localized in proximal tubules, whereas π-GST is found in distal tubules, enabling more precise localization of tubular injury. However, current GST detection relies on ELISA, a time-consuming assay that precludes real-time decision-making, and there has been limited investigation into the distinct GST isoforms and their respective diagnostic thresholds.

In preclinical studies, N-acetyl-β-glucosaminidase (NAG) and kidney injury molecule-1 (KIM-1) have shown potential as predictive biomarkers. In a porcine model using whole blood during 4-h NMP of slaughterhouse-derived kidneys, rapid increases in plasma NAG and IL-6 levels correlated positively with poor gross kidney appearance at 4 h post-perfusion, suggesting that an early, robust inflammatory response is associated with inferior organ quality.^65^ However, because the kidneys were obtained from a slaughterhouse, data on warm ischemia duration were inconsistent or incomplete. Furthermore, although kidney quality was assessed, no transplantation was performed to validate functional outcomes. Future studies should define clinically relevant thresholds for NAG and IL-6 and validate their utility in human transplantation. In an *ex vivo* acellular SNMP system using discarded human kidneys perfused for 4–8 days, LDH, NGAL, and KIM-1 levels in perfusate rose markedly after day 4, coinciding with reduced oxygen consumption, lactate accumulation, and the emergence of an abnormal lipid phenotype in proximal tubules, suggesting that day 4 marks a critical inflection point in the injury trajectory.^66^ Notably, this study captured the dynamic evolution of these biomarkers over prolonged MP rather than at a single time point. However, it lacked transplantation validation, established thresholds, and utilized MP equipment that is complex and not widely accessible. Moving forward, integrating the temporal patterns of LDH, NGAL, and KIM-1 with post-transplant outcomes and defining actionable thresholds could enhance their utility in clinical decision-making.

##### Other biomarkers

The endocrine function of the kidney can also be assessed during MP and may have predictive value. In DCD kidney transplantation using NMP, active perfusate vitamin D levels were significantly higher in grafts that developed DGF postoperatively, whereas urinary urodilatin release was markedly reduced. The rate of urodilatin excretion correlated negatively with DGF duration and positively with urine output, while perfusate EPO release correlated positively with serum creatinine levels 1 week after transplantation.^67^ This study provides a novel perspective on renal function assessment based on the kidney’s endocrine activity.

High-throughput technologies offer new opportunities for identifying potential biomarkers. Van Leeuwen et al.^68^ performed quantitative proteomic analysis of perfusate and found that proteins involved in the classical complement pathway, lipid metabolism, and cytoskeletal organization were significantly associated with graft outcomes at 1 year post-transplantation. In another randomized controlled trial involving 210 perfusate samples, 1,716 proteins were identified; high-density lipoproteins and complement proteins were linked to 12-month graft outcomes, whereas blood-derived proteins were enriched in perfusate from recipients who experienced acute rejection, suggesting that proteomics can help uncover the biological mechanisms underlying MP.^69^ However, metabolomics may have limited predictive utility: a study analyzing 72 metabolites in perfusate found no significant association with transplant outcomes,^70^ indicating that the predictive value of metabolomics in renal MP requires further validation.

Relying solely on perfusion parameters for graft assessment has limitations. Werenski et al.^71^ compared outcomes between the “poor perfusion parameter” (PPP: post-perfusion flow rate ≤80 mL/min and resistance ≥0.40 mmHg mL^−1^ min^−1^) and “optimal perfusion parameter” (OPP) groups across 689 kidney transplants. Short-term graft survival was similar in the PPP and OPP groups (91% vs. 92%), but the PPP group had an increased risk of long-term graft loss (HR = 1.63). Similarly, Qiao et al.^64^ noted that exclusive reliance on perfusion parameters may lead to the unnecessary discard of viable kidneys and should therefore be complemented with additional biomarkers.

The detection of biomarkers during MP can also be combined with non-invasive imaging techniques. The study explores^31^ P magnetic resonance imaging (pMRI) as a noninvasive tool to assess ATP levels and perfusion in donor kidneys after circulatory death, addressing the critical need for reliable viability markers. Experiments in porcine kidneys demonstrated that longer warm ischemia time reduces both ATP production and perfusion, and these reductions inversely correlate with histological injury. These findings suggest that pMRI could aid in predicting graft survival and potentially expand the use of marginal kidneys for transplantation.^72^

#### Heart

In clinical research, lactate also plays an important role as a biomarker of metabolism in heart transplantation during MP. In a multicenter clinical study, a lactate level of less than 5 mmol/L at the end of MP was used as the criterion for transplantability.^73^ In another study, lactate levels at the end of MP were found to be associated with graft failure at 30 days post-operation (specificity = 0.975, sensitivity = 0.625).^74^ However, further research is required to determine the predictive value of this threshold in both DCD and DBD donors. Lactate, as a widely used rapid diagnostic biomarker in clinical practice, holds considerable potential for application. However, there is currently insufficient research on the assessment of lactate during MP in heart transplantation. Future research could focus on monitoring trends in lactate levels to assess the metabolic function of cardiomyocytes and on further determining the cutoff value.

In finding potential biomarkers, a study using a porcine DCD model applied chemical assays and nuclear magnetic resonance (NMR) spectroscopy to identify potential biomarkers in perfusate and blood samples. The results indicated that these biomarkers provide a rapid, multifaceted assessment of graft quality during *ex situ* heart perfusion (ESHP), thereby improving the precision of clinical decisions regarding transplant suitability by offering insights into mitochondrial integrity, energy metabolism, and overall tissue injury.^75^ However, these findings require validation in human trials due to limitations including small sample size and the absence of brain injury, a key feature of human DCD, in the animal model.

#### Lung

In *ex vivo* lung perfusion (EVLP), biomarkers studied include cytokines and molecules associated with cell death and epithelial function.^76^ However, detection methods for these biomarkers—such as ELISA—are not suitable for providing clinicians with timely guidance on whether a transplant can be performed. Therefore, developing rapid and timely detection methods for these molecules and cytokines would facilitate the use of these biomarkers in bedside applications. The release of pro-interleukin-1β (pro-IL-1β) reflects cellular necrosis of donor lung and is a key prognostic biomarker for determining the failure of donor repair.^77^ Elevated levels of epithelial cell death and necrosis markers, such as high-mobility group box-1 (HMGB-1), in EVLP perfusate strongly correlate with declined donor lungs and the subsequent development of severe primary graft dysfunction (PGD).^78^^,^^79^ The increased release of cfDNA, including both nuclear and mtDNA, serves as a significant early predictor of poor graft outcomes.^80^

In EVLP, PGD is commonly used as a clinical outcome measure. However, due to variations in its definition and its reliance on subjective chest X-ray assessments, it has limitations as a prognostic endpoint. At the same time, the predictive value of a single biomarker is limited, and interpreting multiple biomarkers also presents challenges for clinicians. By integrating special inflammatory cytokines (IL-1β, IL-6, IL-8, and IL-10) with physiological measurements and biochemical data, a machine learning framework was established to predict EVLP outcomes.^81^ In a cohort of 338 cases, Sage et al.^82^ constructed the Toronto lung score (TLS_2_) by evaluating IL-6 and IL-8 levels in VELP perfusate. TLS_2_ can predict the decision to transplant at the end of EVLP (AUROC 68%). In one study, machine learning analysis identified five mitochondria-related genes, *MTERF1*, *ACACA*, *COX15*, *OSGEPL1*, and *COQ9*, which were co-regulated during EVLP and ischemia-reperfusion (I/R) in lung transplantation, positioning them as key biomarkers. These findings provide a new approach to the combined analysis of multiple biomarkers.^83^

#### Pancreas

During HMP, pancreatic graft evaluation integrates perfusion parameter with biochemical and tissue biomarkers. Amylase, lipase, LDH, CPK, succinate, and tissue oxygenation have been investigated as indicators of graft injury, metabolism, and function, with tissue succinate and CPK showing potential for predicting ischemic injury.^84^^,^^85^

In NMP, insulin levels in perfusate and glucose tolerance assessed by intravenous glucose tolerance test (IVGTT) in blood samples both demonstrate potential predictive value for evaluating islet cell function.^86^^,^^87^ In oxygenated MP, the oxygen extraction ratio is routinely monitored to assess tissue oxygen utilization.

Pancreatic edema is an important indicator of graft quality, while histological immunohistochemical analyses can provide additional information on islet cell function, cellular injury, and vascularization.^23^^,^^87^^,^^88^ Immunohistochemistry may be employed to evaluate pancreatic tissue for islet cell function, cellular damage, and vascularization by staining with antibodies against insulin, glucagon, somatostatin, caspase, and CD31.^87^^,^^89^

Given the surgical complexity and risks of pancreatic transplantation, clinical studies of pancreatic MP remain unavailable. Preclinical studies, however, suggest that MP may facilitate pancreatic preservation and repair, highlighting the need to identify and validate biomarkers for pancreatic graft assessment.

### Cross-organ biomarkers

The preceding section summarizes recent advances in the identification of potential biomarkers during MP for the liver, kidneys, heart, lungs, and pancreas. Despite organ-specific differences, cross-organ comparison may reveal shared and organ-specific biological signatures (Table 3). When assessing whether an organ is suitable for transplantation, the evaluation can be carried out from three aspects: injury, metabolism, and function.

**Table 3:** Biomarkers of machine perfusion cross organs

Injury biomarkers		Metabolic biomarkers		Functional biomarkers	
Biomarkers	Organ	Biomarkers	Organ	Biomarkers	Organ
Perfusate FMN	LT, NMP/HOPE	perfusate lactate	LT, NMP/HOPE-NMP	bile pH, glucose, HCO_3_^−^, GSH	LT, NMP
Perfusate GST, NGAL, LDH	RT, HMP	perfusate succinate	HT, NMP	urine urodilatin	RT, NMP
Perfusate hypoxanthine, succinate	HT, NMP	^31^P MRI	porcine RT, HMP	perfusate EPO, vitamin D	RT, NMP
Perfusate LDH, succinate, CPK	PT, HMP	tissue succinate	porcine PT, HMP	perfusate insulin, blood glucose	PT, NMP

FMN, flavin mononucleotide; GST, glutathione S-transferase; NGAL, gelatinase-associated lipocalin; LDH, lactate dehydrogenase; CPK, creatine phosphokinase; ^31^P MRI, ^31^P magnetic resonance imaging; GSH, glutathione; EPO, erythropoietin; LT, liver transplantation; HT, heart transplantation; PT, pancreas transplantation; RT, renal transplantation; NMP, normothermic machine perfusion; HMP, hypothermic machine perfusion; HOPE, hypothermic oxygenated machine perfusion.

#### Biomarkers of injury

Injury biomarkers represent the most extensively studied and best-evidenced category among MP biomarkers. An ideal injury biomarker should provide early warning, exhibit cell-type specificity, and correlate with graft loss or serious complications.

In LT, FMN is currently the most extensively studied biomarker of specific damage. It has been validated in both HOPE and NMP studies,^48^^,^^50^^,^^51^ and clinical thresholds have already been established.^15^ In RT, injury markers such as GST and NGAL have been validated,^64^ and LDH showed an AUC of 0.846 for predicting PNF.^63^ Perfusate mt-cfDNA also shows potential but has not yet been validated in clinical practice.^57^ However, unlike FMN in LT, no renal biomarker has been validated in multicenter studies with clearly defined cutoff values.

Hemoglobin, a protein in red blood cells responsible for oxygen transport, has been investigated as an injury marker.^90^ In LT, perfusate hemoglobin during the first 15 min of NMP correlated with transaminase levels, a recognized indicator of injury.^60^ In kidney MP, oxygen carriers are added to the perfusate to reduce tissue injury, which also introduces hemoglobin into the circuit.^91^ Consequently, perfusate hemoglobin reflects not only organ-specific activity but also perfusate composition, particularly when red blood cells are used as an oxygen carrier, limiting its utility as a specific injury biomarker in such settings.

In MP studies of the heart and pancreas, succinic acid has been detected as a marker of ischemic injury.^75^^,^^84^ LDH is also measured during pancreatic MP,^84^^,^^85^ but its organ specificity is considerably lower than that of FMN or GST.

#### Metabolic biomarkers

Metabolic biomarkers directly reflect a graft’s capacity to restore energy metabolism during MP and represent the earliest and most dynamic indicators of viability. Unlike injury or functional biomarkers, they capture the transition from ischemic metabolic suppression to reactivation upon reperfusion.

Lactate clearance has been widely validated as a key indicator of metabolic vitality in the liver. Reported thresholds vary across studies, with observation periods ranging from 2 to 6 h.^38^^,^^39^^,^^44^^,^^92^ The predictive value of lactate plays a significant role in heart transplantation. However, lactate has not demonstrated equivalent predictive value in kidney MP, primarily because it is predominantly metabolized by the liver; thus, renal impairment has a far smaller impact on systemic lactate levels than hepatic dysfunction. In RT, lactate is therefore used only as an auxiliary indicator of perfusion quality, and no clear thresholds exist for predicting DGF or PNF.^65^ This underscores that metabolic biomarker selection should align with the organ’s primary role in systemic metabolism. In porcine kidney transplantation,^31^ P MRI has been used to detect ATP levels.^72^ This technique, which quantifies high-energy phosphate metabolites, shows promise for pre-transplant kidney quality assessment. Other imaging modalities are also employed for graft evaluation. Contrast-enhanced ultrasound can assess donor liver parenchymal perfusion, steatosis, and fibrosis,^93^ and its application during MP warrants further investigation. Dynamic lactate profiling exhibits strong predictive value in LT^40^ and should be explored in MP studies of the kidneys, heart, lungs, and pancreas to clarify the relationship between metabolic biomarker trends and postoperative outcomes.

In addition to lactate, succinate accumulates during tricarboxylic acid cycle disruption and drives reactive oxygen species production. In the DCD myocardial ischemia-reperfusion model, succinate levels in the perfusate were significantly correlated with the extent of ischemic damage.^75^ In pancreas MP, tissue succinate levels were lower than those in the SCS group.^84^ Although succinate has been detected in liver perfusate,^50^ its predictive value has not been systematically evaluated, warranting further investigation in MP.

#### Functional biomarkers

Functional biomarkers reflect an organ’s capacity to perform its physiological functions, with functional restoration being the ultimate goal of transplantation. In the liver, bile, a liver-specific product, provides a unique measure of organ function. Because cholangiocytes actively secrete bicarbonate and reabsorb glucose, bile bicarbonate concentration, pH, and glucose levels serve as useful indicators of biliary tract function.^53^ Moreover, a dynamic increase in biliary GSH levels is associated with a lower risk of biliary complications.^56^ In the kidneys, urine and endocrine molecules are primary sources of functional biomarkers; for instance, the urinary urodilatin excretion rate is negatively correlated with the duration of DGF and positively correlated with urine output, directly reflecting the renal tubules’ responsiveness to volume load.^67^ Glucose and sodium reabsorption can be maintained during SNMP,^66^ indicating preserved proximal tubule transport function. However, clinical application of urine- and bile-based biomarkers is limited by the possibility that these fluids may not be produced. Therefore, the selection of functional biomarkers should prioritize those that are stably produced under MP conditions and amenable to straightforward detection. As direct products of liver and kidney function, bile and urine warrant further in-depth investigation.

## Challenges and future directions for clinical translation

### Challenge

#### Lack of standardized sampling, assays, and cutoff values

A major challenge is how to validate and standardize biomarkers. First, the heterogeneity of the data makes comparison difficult. Samples from various sources in each study included perfusate, blood, bile, and urine. Differences in perfusion duration also led to variation in sampling time points. The absence of standardized sampling time points complicates comparisons, as biomarker levels may change substantially during perfusion. Although dynamic monitoring provides information on biomarker kinetics, its implementation is challenged by differences in optimal sampling frequency, biomarker kinetics, and perfusate composition.

Moreover, monitoring ways of the same biomarkers varied between existing trails, which led to different thresholds. For example, perfusate FMN quantified by fluorimetry or LC-MS have different thresholds. Not only that, even though special cfDNA,^58^^,^^59^ sPD-1,^62^ NAG,^65^ and IL-6^65^ biomarkers have been proved the relevance of organ quality, detecting those biomarkers takes more time. If those biomarkers are used as prospective research variables, the *ex vivo* time of organs would be artificially prolonged.

Reported cutoff values for lactate level vary across studies, ranging from <2.5 to <4.1 mmol/L,^39^^,^^41^^,^^42^ and the threshold of FMN varies from <10.6 ng/mL^15^ to <5,165 a.u.^48^ Such heterogeneity may lead to inconsistent clinical decisions, with the same lactate value potentially supporting transplantation in one study but organ discard in another.

When using biomarkers to guide clinical decision-making, standardized monitoring time, testing methods, and defined cutoffs can lend greater credibility to clinical decisions.

#### Limited prospective clinical evidence

Most studies of MP biomarkers remain correlational, with a persistent lack of direct investigations into the specificity and sensitivity. Studies of biomarkers in MP are still in their early stages, even though an increasing number of clinical trials focus on MP in liver, kidney, heart, and lung transplantation.

Although ongoing clinical trials (listed in Table 4) include multicenter, prospective studies evaluating the efficacy of MP, few directly investigate biomarkers. Multicenter-validated cutoffs for biomarker-guided decision-making are also lacking. Most biomarker research in LT focuses on NMP or treats biomarkers as secondary endpoints rather than primary validation targets. Nevertheless, ongoing studies can facilitate standardized collection of perfusate, bile, and urine for biomarker validation. Samples collected at predefined time points must be accompanied by detailed documentation of pre-processing and storage protocols. Existing trials enroll diverse donor types, including DCD, ECD, DBD, and discarded organs, providing a broad spectrum for assessing the utility of MP. Clinically relevant endpoints and systematic long-term follow-up are also required. Large-scale, prospective, organ-specific cohort studies are further needed to validate the sensitivity, specificity, and predictive performance of candidate biomarkers.

**Table 4:** Ongoing clinical trials on biomarkers or organ assessment in machine perfusion

Trial ID	Organ	Perfusion	Direct biomarker study	Biomarkers	Endpoint
NCT04744389 Multicenter, prospective, randomized study	liver	HMP, NMP	no	perfusate HMGB1	graft loss
NCT04619732 Pilot study	kidney	HMP	no	creatine, glucose, lactate	post-operative recovery of kidney function
NCT05951231 Single center, prospective, open-label cohort study	liver	NMP	no	lactate level in microdialysate	lactate level in microdialysate from hilar plate after 4 h NMP
NCT06950398 Multicenter, prospective study	liver	NMP	no, organ quality evaluation	not mentioned	no explicit endpoint
NCT06371924 Randomized study	liver	NMP	no, organ quality evaluation	mitochondrial complex I enzyme activity in liver tissue samples	no explicit endpoint
NCT03456284 Single center prospective cohort pilot study	liver	NMP	no, organ quality evaluation	not mentioned	patient survival rate after 30 days

MP, machine perfusion; NMP, normothermic perfusion; HMP, hypothermic perfusion; HMGB1, high-mobility group box 1.

#### Heterogeneity across donors, organs, and MP protocols

The physiological differences among donors and organs lead to variable baseline characteristics, making it difficult to apply a uniform assessment standard. This heterogeneity directly affects biomarker interpretation and clinical utility. In RT, donor age and comorbidities such as hypertension and diabetes can influence graft outcomes and pathological findings.^94^^,^^95^^,^^96^ Similarly, perfusate FMN levels differ between DCD and DBD livers and between discarded and transplanted grafts.^48^ The substantial heterogeneity in organ origin and quality remains a core challenge in building a universal biomarker evaluation system. Factors such as donor age, underlying diseases, cause of death, and resuscitation history jointly determine the organ’s initial injury state. These baseline differences can alter graft responses during MP and biomarker kinetics.

Physiological functions, injury mechanisms, and metabolic states differ markedly across organs. A single biomarker cannot be readily applied across organ types. For instance, bile, an indicator of liver function, is not applicable to other organs. Similarly, while lactate is not liver specific, its clinically relevant thresholds and reference ranges vary when used for other organs.

MP system configurations continue to undergo refinement. The incorporation of filtration devices and the use of varying perfusate compositions can all affect biomarker concentrations, metabolic rates, and enzyme activity during MP. Within the perfusion circuit, biomarker concentrations may differ between arterial and venous segments, as well as between pre-filter and post-filter regions; however, few studies specify exact sampling sites. Variations in perfusate composition directly impact biomarker baselines. For instance, in RBC-based systems, ongoing lactate production by erythrocytes confounds lactate clearance as a marker of hepatocyte metabolic recovery. Consequently, biomarker generalizability must be critically validated across diverse perfusion platforms. Furthermore, differences of MP circle’s capacity reduce the comparability of the absolute concentrations of biomarkers. Pump configuration may also affect biomarker levels through shear stress and hemolysis, meaning that changes in lactate, AST, or potassium may not solely reflect graft injury. Differences in the oxygen levels and temperature of the MP equipment can also lead to variations in the results. Biomarkers used to assess organ function at NMP, such as lactate clearance and bile production, show a limited predictive ability at HMP or SNMP, as cellular metabolism is inhibited under such conditions.

### Future directions

#### Co-evolution of perfusion platforms, clinically derived biomarkers, and comprehensive data interpretation

The biomarkers in MP used in clinical purposes demand to improve in three areas: MP equipment, the selection of biomarkers, and the analysis of indicators.

The equipment of MP should be upgraded in the future. Until now, commercially used MP equipment in clinics, such as LifePort, OrganOx, and TransMedics, have disadvantage of being large and inconvenient. Thus, for clinical convenience, compact, portable devices are better suited to clinical use. A multicenter randomized controlled trial has demonstrated the advantages of portable NMP in donors sourced from DCD.^97^ The effectiveness of the portable HOPE system in improving early functional outcomes following LT has also been validated in a multicenter, randomized controlled trial.^98^ Moreover, MP equipment should be capable of automatically sampling and analyzing biomarkers in real time for dynamic monitoring.

Biomarkers should also be selected based on clinical feasibility and rapid detectability. Parameters such as lactate, pH, glucose, and electrolytes can be rapidly measured using routine blood gas analyzers, suggesting the potential for their integration into MP systems. However, differences in perfusate composition, metabolite accumulation, and hemolysis may interfere with measurements, requiring MP-specific calibration and validation.

Rather than relying on individual biomarkers, integrated multi-parameter assessment may provide a more comprehensive evaluation of graft quality. Automated integration of biomarker data with perfusion parameters could support real-time clinical interpretation, while machine-learning models may further assist clinicians in interpreting complex biomarker profiles and predicting graft outcomes.

#### Establishment of clinical validation and standardization

Standardization of MP biomarkers is challenged by differences in perfusion platforms, monitoring parameters, donor characteristics, and clinical outcomes. In the difference of MP equipment, the concentration parameter alone may be affected by variations in absolute concentration resulting from differences in the pre-flush volume of different devices. Normalizing biomarker changes using concentration gradients or biomarker ratios may reduce platform-related variation. The metabolic activity of the *ex vivo* organ can be assessed by calculating its half-life or extraction rate by introducing a specific exogenous metabolic tracer into MP circuit. For MP equipment at different temperatures, distinct biomarker systems can be established to assess organ quality. Most importantly, we need to further advance the development of an expert consensus on MP of different organs.

Common parameters recorded across MP platforms include perfusion time, pressure, and flow rate. Monitoring frequency should be determined by biomarker kinetics, with rapidly changing biomarkers requiring more frequent sampling. Sampling sites should also be standardized according to circuit configuration and clinical context. Multicenter studies should establish standardized protocols for sampling time points, monitoring methods, and clinical outcomes.

Dynamic monitoring may reduce the influence of donor baseline differences on biomarker changes. Future studies should incorporate donor risk stratification across DCD, ECD, and other donor categories. Donor information has predictive value for early post-transplant function.^99^ Large prospective studies should incorporate donor characteristics into biomarker models, as the predictive value of a given threshold may differ across donor subgroups.

Most current studies are small-scale observational studies, and there is still a lack of data on long-term outcomes following MP of organs. Existing studies could further investigate the long-term prognosis of patients who undergo transplantation following perfusion. In the selection of clinical outcomes, different biomarkers vary in their predictive value for long-term or short-term clinical outcomes.

#### From assessment to therapeutic intervention

Biomarker research should not only enable timely detection of changes in organ function but also guide therapeutic interventions. Therefore, it remains critical to clarify whether a biomarker reflects underlying injury or actively contributes to its progression. Biomarkers that contribute to tissue injury may also serve as therapeutic targets. Long-term NMP could extend the preservation time of organs,^21^^,^^22^ thereby providing a window of opportunity for treatment. Moreover, biomarkers can act as real-time indicators of treatment efficacy. For example, after administering mitochondrial protectants via perfusion, one can monitor reductions in FMN release and improvements in lactate clearance. Perfusate serves not only as a source for biomarker detection but also as a vehicle for targeted drug delivery at reduced doses. Bone marrow mesenchymal stem cells combined with MP have demonstrated therapeutic potential in attenuating hepatic sinusoidal endothelial injury in LT.^100^^,^^101^^,^^102^^,^^103^ Similarly, cell-based therapies, such as multipotent adult progenitor cells delivered during NMP in kidney transplantation, have shown the ability to enhance organ quality and reduce IRI prior to transplantation.^104^ In addition, nanoparticles administered during NMP offer a novel strategy to mitigate IRI,^105^ and combining ferroptosis modulators with NMP has shown regulatory effects on IRI and represents a promising treatment approach in LT.^106^ Gene therapy within MP platforms is also under active investigation,^107^^,^^108^ and preclinical studies have validated drug delivery via NMP as a novel therapeutic strategy.^109^ Future work should focus on integrating real-time biomarker monitoring during MP with emerging therapeutic modalities, thereby transforming this technology from a passive organ preservation system into an active platform for repair and regeneration. If, following assessment, an organ is deemed unsuitable for transplantation, therapeutic measures may be taken to repair the damage, after which the feasibility of transplantation can be reassessed.

#### Biomarker-guided clinical decision-making

Research into biomarkers in MP is not only about identifying new biomarkers, but, more importantly, about guiding clinical decision-making. For example, biomarkers in liver MP can be combined with multiple parameters—such as lactate, FMN, transaminases, bile characteristics, and perfusion parameters—to comprehensively assess liver metabolism, function, and cellular injury. Although organs undergo processes of tissue repair and metabolic function while outside the body, the types of biomarkers vary between different organs. Clinicians also need to interpret the results of biomarker tests and, considering the donor’s individual circumstances, changes in biomarker levels, and the results of the organ pathology examination, carry out a comprehensive assessment of the organ. Doctors should stratify donors prior to assessment, as the rate at which lactate is cleared during lactate metabolism differs between the liver of a young donor and that of an elderly patient with fatty liver disease. Organs from ECD donors differ from those from DCD donors at baseline; therefore, attention should be paid to the dynamic trends in biomarkers while assessing the recovery of organ function. Organ assessment requires a small set of rapid, simple, and sensitive indicators of cellular injury. For example, in the case of lactate in the liver, if lactate levels do not fall within a certain range within a specified period of perfusion, clinicians should decide whether the organ requires continued perfusion or whether it is suitable for transplantation. However, a single indicator tends to reflect only a part of an organ; it should therefore be considered in conjunction with other indicators, such as those that reflect metabolism and function and those that indicate whether the organ can function normally when isolated from the body. Core biomarkers serve as the primary basis for assessing organ quality, while supplementary biomarkers can be used as indicators to determine whether perfusion should continue. If core biomarkers fall below validated thresholds and metabolic and functional biomarkers show favorable trends, the graft may be considered suitable for transplantation. If the core parameters remain above the threshold but other parameters reflecting metabolism and function are gradually rising, the perfusion time may be extended or intervention measures taken, followed by a reassessment of organ quality after the intervention. If the trends in multiple core parameters of the organ are deteriorating and intervention measures prove ineffective, the organ should be discarded following a thorough assessment.

Furthermore, in clinical practice, biomarkers can be combined with histopathological findings. The results of a zero-hour pathological biopsy provide irreversible baseline indicators for assessing organ quality. Biomarkers, on the other hand, can reflect the processes of tissue repair and functional metabolism in the organs during subsequent MP. When pathology indicates substantial injury, but dynamic MP biomarkers show improving metabolic and functional profiles, the graft may retain reparative potential and could become suitable for transplantation after intervention or prolonged perfusion. For kidneys borderline for single- versus dual-kidney transplantation based on Remuzzi or Banff scoring, dynamic MP assessment may identify grafts with recovery potential, enabling targeted intervention and potentially increasing organ utilization.

Despite the potential of MP biomarkers for clinical decision-making, their clinical validation and mechanistic understanding remain limited. Large prospective studies are needed to validate biomarker performance and clarify their biological roles. The goal is to develop biomarker-driven, individualized evaluation and intervention strategies that improve transplant outcomes for marginal donors, thereby expanding the pool of usable organs and alleviating the supply-demand imbalance (Figure 4).

**Figure 4:**
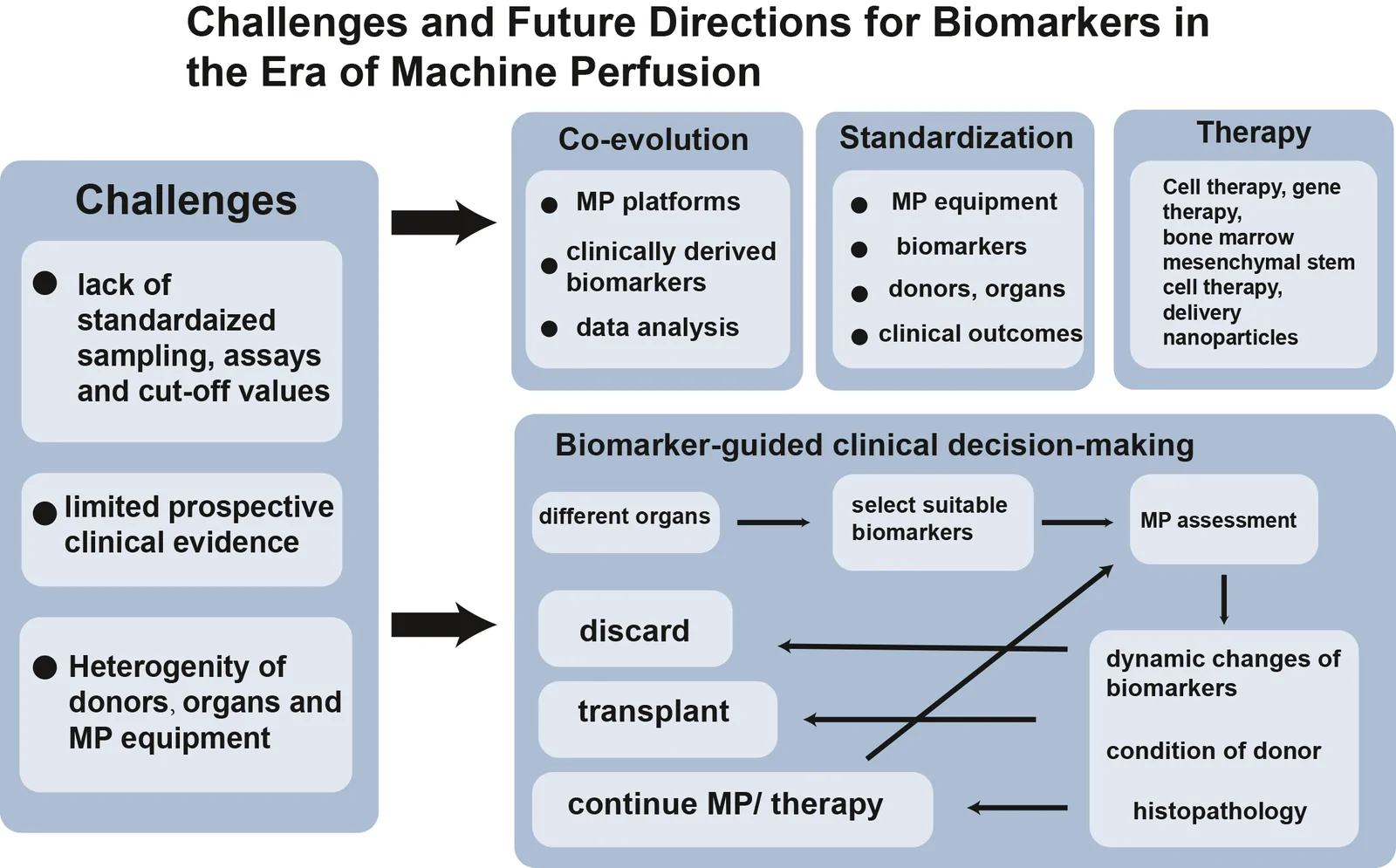
Challenges and future directions for biomarkers in machine perfusion Clinical translation is limited by insufficient standardization, prospective validation, and heterogeneity across MP equipment, organs, and donor characteristics. Standardization and the co-evolution of MP technologies, biomarker analysis, and clinical validation are essential. MP also provides a platform for therapeutic intervention. Biomarker-guided clinical decision-making integrates biomarker dynamics, donor characteristics, and histopathology for comprehensive graft assessment.

### Conclusions

Dynamic biomarkers hold substantial predictive value for organ preservation and evaluation. MP has evolved through a prolonged developmental process and continues to undergo refinement. In contrast to SCS, MP has transitioned from passively suppressing metabolism to actively recapitulating physiological perfusion conditions. As a result, it enables dynamic, real-time, and rapid assessment of organ quality. This capability necessitates specific and sensitive biomarkers to serve as indicators for monitoring and reflecting organ status, thereby facilitating early intervention for organ injury, supporting post-transplant functional recovery, and predicting the development of complications. Clinical and preclinical data on potential biomarkers are available for liver and kidney transplantation. However, current research remains largely in the discovery phase, with insufficient analytical and clinical validation. Substantial progress is required before regulatory approval and routine clinical implementation can be realized. Studies in pancreatic, cardiac, and lung transplantation remain preclinical, primarily focused on establishing the feasibility and reproducibility of MP. Future studies should prioritize biomarkers with strong reproducibility, biological relevance, and clinical feasibility, while establishing standardized thresholds through prospective multicenter validation. Rather than relying on individual biomarkers, integrated panels combining complementary indicators with perfusion parameters and histological assessment may provide a more robust framework for evaluating graft quality. Dynamic changes in biomarker levels during perfusion may further enable real-time assessment and intervention. Ultimately, biomarker-guided MP has the potential to move organ assessment beyond static evaluation toward individualized, data-driven decision-making and more effective utilization of marginal grafts. We believe that precision organ protection strategies will enhance the utilization of marginal donors, improve graft function, increase long-term survival rates, and ultimately expand the population benefiting from transplantation.

## Acknowledgments

This work was supported by grants from the Transplantation Pioneer Program of the China Organ Transplantation Development Foundation (no. YZLC-2025-004) and the Shanxi Province Basic Research Program (grant no. 202203021211079).

## Author contributions

Conceptualization, methodology, investigation, formal analysis, and writing – original draft, Z.Y.; data curation, X.Y.; visualization, investigation, and funding acquisition, Z.G.; resources and supervision, S.D.; project administration, Y.Z.; writing – review & editing, H.S.; conceptualization, funding acquisition, resources, supervision, and writing – review & editing, J.J.

## Declaration of interests

The authors declare no competing interests.

## References

[1] Pezzati D., Torri F., Franzini M., Balzano E., Catalano G., Tincani G., Bronzoni J., Martinelli C., Trizzino A., Petagna L. (2025). Association of perfusate cytokine concentrations during liver graft ex situ normothermic perfusion to donor type and postoperative outcomes. Liver Transplant..

[2] Pradat P., Pantel S., Maynard M., Lalande L., Thevenon S., Adam R., Allard M.A., Robin F., Rayar M., Boleslawski E. (2023). End-ischemic hypothermic oxygenated perfusion for extended criteria donors in liver transplantation: a multicenter, randomized controlled trial-HOPExt. Trials.

[3] Yoshida K., Nakamura S., Sakamoto H., Kondo M., Chouno T., Ikegami Y., Shirakigawa N., Mizumoto H., Yamashita Y.I., Baba H., Ijima H. (2021). Normothermic machine perfusion system satisfying oxygen demand of liver could maintain liver function more than subnormothermic machine perfusion. J. Biosci. Bioeng..

[4] Thorne A.M., Geng Y., Lantinga V.A., Smit M., Kuivenhoven J.A., Porte R.J., Kuipers F., Olinga P., Wolters J.C., de Meijer V.E. (2025). Therapeutic hyperthermia promotes lipid export and HSP70/90 during machine perfusion of human livers. Phys. Rep..

[5] Tan X.Y., Kuang W.J., Deng F.W., Huang Z.P., Ouyang Q., Huang X.T., Ho W.I., Liang M.J., Huo F., Chen H.W. (2025). Six-hour local 4 °C dual hypothermic oxygenated machine perfusion improves the preservation of porcine liver after cardiac death using an ex vivo reperfusion model. Hepatobiliary Pancreat. Dis. Int..

[6] Lau N.S., Ly M., Dennis C., Toomath S., Huang J.L., Huang J., Ly H., Chanda S., Marinelli T., Davis R. (2024). Microbial Contamination During Long-term Ex Vivo Normothermic Machine Perfusion of Human Livers. Transplantation.

[7] Puttappa A., Gaurav R., Kakhandki V., Swift L., Fear C., Webster R., Radwan A., Mohammed M., Butler A., Klinck J., Watson C. (2025). Normothermic regional and ex situ perfusion reduces postreperfusion syndrome in donation after circulatory death liver transplantation: A retrospective comparative study. Am. J. Transplant..

[8] Lascaris B., Bodewes S.B., Thorne A.M., van den Heuvel M.C., de Haas R.J., Nijsten M.W.N., de Meijer V.E., Porte R.J. (2025). Perfusion Pressures and Weight Loss During Normothermic Machine Perfusion of Human Donor Livers. Artif. Organs.

[9] Hosgood S.A., Callaghan C.J., Wilson C.H., Smith L., Mullings J., Mehew J., Oniscu G.C., Phillips B.L., Bates L., Nicholson M.L. (2023). Normothermic machine perfusion versus static cold storage in donation after circulatory death kidney transplantation: a randomized controlled trial. Nat. Med..

[10] Shamaa T.M., Shamaa O., Crombez C., Konel J.M., Kitajima T., Shimada S., Ivanics T., Mohamed A., Collins K., Nagai S. (2022). The use of normothermic liver preservation in combined liver and lung transplantation: A single-center experience. Am. J. Transplant..

[11] Sponga S., Vendramin I., Salman J., Ferrara V., De Manna N.D., Lechiancole A., Warnecke G., Dralov A., Haverich A., Ius F. (2023). Heart Transplantation in High-Risk Recipients Employing Donor Marginal Grafts Preserved With Ex-Vivo Perfusion. Transpl. Int..

[12] Califf R.M. (2018). Biomarker definitions and their applications. Exp. Biol. Med..

[13] Dumbill R., Knight S., Hunter J., Fallon J., Voyce D., Barrett J., Ellen M., Conroy E., Roberts I.S., James T. (2025). Prolonged normothermic perfusion of the kidney prior to transplantation: a historically controlled, phase 1 cohort study. Nat. Commun..

[14] Hautz T., Hackl H., Gottschling H., Gronauer R., Hofmann J., Salcher S., Zelger B., Oberhuber R., Cardini B., Weissenbacher A. (2024). Transcriptomic signatures during normothermic liver machine perfusion correspond with graft quality and predict the early graft function. EBioMedicine.

[15] Dingfelder J., Kollmann D., Rauter L., Pereyra D., Kacar S., Weijler A.M., Saffarian Zadeh T., Tortopis C., Silberhumer G., Salat A. (2025). Validation of mitochondrial FMN as a predictor for early allograft dysfunction and patient survival measured during hypothermic oxygenated perfusion. Liver Transplant..

[16] Martins P.N., Buchwald J.E., Mergental H., Vargas L., Quintini C. (2020). The role of normothermic machine perfusion in liver transplantation. Int. J. Surg..

[17] Dutkowski P., de Rougemont O., Clavien P.A. (2008). Alexis Carrel: genius, innovator and ideologist. Am. J. Transplant..

[18] Sousa Da Silva R.X., Weber A., Dutkowski P., Clavien P.A. (2022). Machine perfusion in liver transplantation. Hepatology.

[19] Belzer F.O., Ashby B.S., Gulyassy P.F., Powell M. (1968). Successful seventeen-hour preservation and transplantation of human-cadaver kidney. N. Engl. J. Med..

[20] Lau N.S., McCaughan G., Ly M., Liu K., Crawford M., Pulitano C. (2024). Long-term machine perfusion of human split livers: a new model for regenerative and translational research. Nat. Commun..

[21] Lau N.S., Ly M., Dennis C., Jacques A., Cabanes-Creus M., Toomath S., Huang J., Mestrovic N., Yousif P., Chanda S. (2023). Long-term ex situ normothermic perfusion of human split livers for more than 1 week. Nat. Commun..

[22] Ly M., Lau N.S., Dennis C., Chen J., Risbey C., Tan S., Chen R., Wang C., Gorrell M.D., McKenzie C. (2025). Long-term ex situ normothermic machine perfusion allows regeneration of human livers with severe bile duct injury. Am. J. Transplant..

[23] Doppenberg J.B., Leemkuil M., Engelse M.A., Krikke C., de Koning E.J.P., Leuvenink H.G.D. (2021). Hypothermic oxygenated machine perfusion of the human pancreas for clinical islet isolation: a prospective feasibility study. Transpl. Int..

[24] Fu Y., Wang Y., Liang L., Gu M., Gao Y., Feng L. (2023). Mesenchymal Stem Cell Utilization for In Vitro Donor Liver Machine Perfusion Preservation: Current Status and Future Directions. Stem Cells Transl. Med..

[25] López-Martínez S., Simón C., Santamaria X. (2024). Normothermic Machine Perfusion Systems: Where Do We Go From Here?. Transplantation.

[26] Resch T., Cardini B., Oberhuber R., Weissenbacher A., Dumfarth J., Krapf C., Boesmueller C., Oefner D., Grimm M., Schneeberger S. (2020). Transplanting Marginal Organs in the Era of Modern Machine Perfusion and Advanced Organ Monitoring. Front. Immunol..

[27] Mazilescu L.I., Parmentier C., Kalimuthu S.N., Ganesh S., Kawamura M., Goto T., Noguchi Y., Selzner M., Reichman T.W. (2022). Normothermic ex situ pancreas perfusion for the preservation of porcine pancreas grafts. Am. J. Transplant..

[28] Puntmann V.O. (2009). How-to guide on biomarkers: biomarker definitions, validation and applications with examples from cardiovascular disease. Postgrad. Med. J..

[29] Guo L., Jia L., Luo L., Xu X., Xiang Y., Ren Y., Ren D., Shen L., Liang T. (2022). Critical Roles of Circular RNA in Tumor Metastasis via Acting as a Sponge of miRNA/isomiR. Int. J. Mol. Sci..

[30] Zhang Z., Yang T., Xiao J. (2018). Circular RNAs: Promising Biomarkers for Human Diseases. EBioMedicine.

[31] de Fraipont F., Gazzeri S., Cho W.C., Eymin B. (2019). Circular RNAs and RNA Splice Variants as Biomarkers for Prognosis and Therapeutic Response in the Liquid Biopsies of Lung Cancer Patients. Front. Genet..

[32] Olthoff K.M., Kulik L., Samstein B., Kaminski M., Abecassis M., Emond J., Shaked A., Christie J.D. (2010). Validation of a current definition of early allograft dysfunction in liver transplant recipients and analysis of risk factors. Liver Transplant..

[33] Pareja E., Cortes M., Hervás D., Mir J., Valdivieso A., Castell J.V., Lahoz A. (2015). A score model for the continuous grading of early allograft dysfunction severity. Liver Transplant..

[34] Agopian V.G., Harlander-Locke M.P., Markovic D., Dumronggittigule W., Xia V., Kaldas F.M., Zarrinpar A., Yersiz H., Farmer D.G., Hiatt J.R., Busuttil R.W. (2018). Evaluation of Early Allograft Function Using the Liver Graft Assessment Following Transplantation Risk Score Model. JAMA Surg..

[35] Avolio A.W., Franco A., Schlegel A., Lai Q., Meli S., Burra P., Patrono D., Ravaioli M., Bassi D., Ferla F. (2020). Development and Validation of a Comprehensive Model to Estimate Early Allograft Failure Among Patients Requiring Early Liver Retransplant. JAMA Surg..

[36] Meszaros A.T., Hofmann J., Buch M.L., Cardini B., Dunzendorfer-Matt T., Nardin F., Blumer M.J., Fodor M., Hermann M., Zelger B. (2022). Mitochondrial respiration during normothermic liver machine perfusion predicts clinical outcome. EBioMedicine.

[37] Mergental H., Laing R.W., Kirkham A.J., Perera M.T.P.R., Boteon Y.L., Attard J., Barton D., Curbishley S., Wilkhu M., Neil D.A.H. (2020). Transplantation of discarded livers following viability testing with normothermic machine perfusion. Nat. Commun..

[38] Liu Q., Del Prete L., Ali K., Grady P., Bilancini M., Etterling J., D’Amico G., Diago Uso T., Hashimoto K., Aucejo F. (2023). Sequential hypothermic and normothermic perfusion preservation and transplantation of expanded criteria donor livers. Surgery.

[39] Quintini C., Del Prete L., Simioni A., Del Angel L., Diago Uso T., D'Amico G., Hashimoto K., Aucejo F., Fujiki M., Eghtesad B. (2022). Transplantation of declined livers after normothermic perfusion. Surgery.

[40] Hofmann J., Meszaros A.T., Butler A., Hann A., Hartog H., Kneifel F., Iype S., Crick K., Cardini B., Fiore B. (2024). Predictive value of early postoperative lactate (<6 h) during normothermic machine perfusion and outcome after liver transplantation: results from a multicentre study. Br. J. Surg..

[41] Mergental H., Stephenson B.T.F., Laing R.W., Kirkham A.J., Neil D.A.H., Wallace L.L., Boteon Y.L., Widmer J., Bhogal R.H., Perera M.T.P.R. (2018). Development of Clinical Criteria for Functional Assessment to Predict Primary Nonfunction of High-Risk Livers Using Normothermic Machine Perfusion. Liver Transplant..

[42] Mergental H., Perera M.T.P.R., Laing R.W., Muiesan P., Isaac J.R., Smith A., Stephenson B.T.F., Cilliers H., Neil D.A.H., Hübscher S.G. (2016). Transplantation of Declined Liver Allografts Following Normothermic Ex-Situ Evaluation. Am. J. Transplant..

[43] Patrono D., De Carlis R., Gambella A., Farnesi F., Podestà A., Lauterio A., Tandoi F., De Carlis L., Romagnoli R. (2023). Viability assessment and transplantation of fatty liver grafts using end-ischemic normothermic machine perfusion. Liver Transplant..

[44] Mergental H., Laing R.W., Hodson J., Boteon Y.L., Attard J.A., Walace L.L., Neil D.A.H., Barton D., Schlegel A., Muiesan P. (2022). Introduction of the Concept of Diagnostic Sensitivity and Specificity of Normothermic Perfusion Protocols to Assess High-Risk Donor Livers. Liver Transplant..

[45] von Horn C., Winzen N., Malkus L., Saner F., Minor T. (2025). Machine Perfusion Parameters to Predict Graft Injury or Early Function After Liver Transplantation. Exp. Clin. Transplant..

[46] Patrono D., Catalano G., Rizza G., Lavorato N., Berchialla P., Gambella A., Caropreso P., Mengozzi G., Romagnoli R. (2020). Perfusate Analysis During Dual Hypothermic Oxygenated Machine Perfusion of Liver Grafts: Correlations With Donor Factors and Early Outcomes. Transplantation.

[47] Watson C.J.E., Gaurav R., Fear C., Swift L., Selves L., Ceresa C.D.L., Upponi S.S., Brais R., Allison M., Macdonald-Wallis C. (2022). Predicting Early Allograft Function After Normothermic Machine Perfusion. Transplantation.

[48] Eden J., Thorne A.M., Bodewes S.B., Patrono D., Roggio D., Breuer E., Lonati C., Dondossola D., Panayotova G., Boteon A.P.C.S. (2025). Assessment of liver graft quality during hypothermic oxygenated perfusion: The first international validation study. J. Hepatol..

[49] Schlegel A., Muller X., Mueller M., Stepanova A., Kron P., de Rougemont O., Muiesan P., Clavien P.A., Galkin A., Meierhofer D., Dutkowski P. (2020). Hypothermic oxygenated perfusion protects from mitochondrial injury before liver transplantation. EBioMedicine.

[50] Muller X., Schlegel A., Kron P., Eshmuminov D., Würdinger M., Meierhofer D., Clavien P.A., Dutkowski P. (2019). Novel Real-time Prediction of Liver Graft Function During Hypothermic Oxygenated Machine Perfusion Before Liver Transplantation. Ann. Surg..

[51] Wehrle C.J., Satish S., Dewey E., Nadeem M.A., Sun K., Jiao C., Khalil M., Pita A., Kim J., Aucejo F. (2025). A New Era of Decision-making in Liver Transplantation: A Prospective Validation and Cost-effectiveness Analysis of FMN-guided Liver Viability Assessment During Normothermic Machine Perfusion. Ann. Surg..

[52] Sun K., Jiao C., Panconesi R., Satish S., Karakaya O.F., De Goeij F.H.C., Diwan T., Ali K., Cadinu L.A., Cazzaniga B. (2025). Quantifying Flavin mononucleotide: an internationally validated methodological approach for enhanced decision making in organ transplantation. EBioMedicine.

[53] Matton A.P.M., de Vries Y., Burlage L.C., van Rijn R., Fujiyoshi M., de Meijer V.E., de Boer M.T., de Kleine R.H.J., Verkade H.J., Gouw A.S.H. (2019). Biliary Bicarbonate, pH, and Glucose Are Suitable Biomarkers of Biliary Viability During Ex Situ Normothermic Machine Perfusion of Human Donor Livers. Transplantation.

[54] van Leeuwen O.B., de Vries Y., Fujiyoshi M., Nijsten M.W.N., Ubbink R., Pelgrim G.J., Werner M.J.M., Reyntjens K.M.E.M., van den Berg A.P., de Boer M.T. (2019). Transplantation of High-risk Donor Livers After Ex Situ Resuscitation and Assessment Using Combined Hypo- and Normothermic Machine Perfusion: A Prospective Clinical Trial. Ann. Surg..

[55] de Jong I.E.M., Bodewes S.B., van Leeuwen O.B., Oosterhuis D., Lantinga V.A., Thorne A.M., Lascaris B., van den Heuvel M.C., Wells R.G., Olinga P. (2023). Restoration of Bile Duct Injury of Donor Livers During Ex Situ Normothermic Machine Perfusion. Transplantation.

[56] Bogensperger C., Esser H., Ponholzer F., Cardini B., Hofmann J., Messner F., Oberhuber R., Resch T., Öfner D., Schneeberger S., Weissenbacher A. (2026). Biliary Glutathione as a Potential Marker for Viability Assessment of Cholangiocytes During Normothermic Machine Perfusion of Livers. Artif. Organs.

[57] Westhaver L.P., Nersesian S., Arseneau R.J., Hefler J., Hargreaves B.K.V., Edgar A., Azizieh Y., Cuesta-Gomez N., Izquierdo D.L., Shapiro A.M.J. (2024). Mitochondrial DNA levels in perfusate and bile during ex vivo normothermic machine correspond with donor liver quality. Heliyon.

[58] Cox D.R.A., Lee E., Wong B.K.L., McClure T., Zhang F., Goh S.K., Vago A., Jackett L., Fink M., Jones R. (2024). Graft-derived cfDNA Monitoring in Plasma and Bile During Normothermic Machine Perfusion in Liver Transplantation Is Feasible and a Potential Tool for Assessing Graft Viability. Transplantation.

[59] Sorbini M., Carradori T., Patrono D., Togliatto G., Caorsi C., Vaisitti T., Mansouri M., Delsedime L., Vissio E., De Stefano N. (2025). Circulating cell-free DNA in liver transplantation: A pre- and post-transplant biomarker of graft dysfunction. Artif. Organs.

[60] Maeda A., Starkey G., Spano S., Chaba A., Eastwood G., Yoshino O., Perini M.V., Fink M., Bellomo R., Jones R. (2025). Perfusate hemoglobin during normothermic liver machine perfusion as biomarker of early allograft dysfunction: A pilot study. Artif. Organs.

[61] Weissenbacher A., Bogensperger C., Oberhuber R., Meszaros A., Gasteiger S., Ulmer H., Berchtold V., Krendl F.J., Fodor M., Messner F. (2022). Perfusate Enzymes and Platelets Indicate Early Allograft Dysfunction After Transplantation of Normothermically Preserved Livers. Transplantation.

[62] Irsara C., Weissenbacher A., Krendl F.J., Anliker M., Hofmann J., Hautz T., Schneeberger S., Griesmacher A., Loacker L. (2025). Expression of sPD-L1 levels in an ex vivo liver perfusion model. Clin. Exp. Immunol..

[63] Leite R.R.d.A., Leite Jr M., Einicker-Lamas M., Valverde R.H.F., Miranda L.C.D., Schanaider A. (2024). Clinical outcomes prediction in kidney transplantation by use of biomarkers from hypothermic machine perfusion. Int. Braz. J. Urol..

[64] Qiao Y., Ding C., Li Y., Tian X., Tian P., Ding X., Xiang H., Zheng J., Xue W. (2021). Predictive value of hypothermic machine perfusion parameters combined perfusate biomarkers in deceased donor kidney transplantation. Chin. Med. J..

[65] Steinhauser C., Yakac A., Markgraf W., Kromnik S., Döcke A., Talhofer P., Thiele C., Malberg H., Sommer U., Baretton G.B. (2024). Assessing Biomarkers of Porcine Kidneys under Normothermic Machine Perfusion-Can We Gain Insight into a Marginal Organ?. Int. J. Mol. Sci..

[66] de Haan M.J.A., Jacobs M.E., Witjas F.M.R., de Graaf A.M.A., Sánchez-López E., Kostidis S., Giera M., Calderon Novoa F., Chu T., Selzner M. (2024). A cell-free nutrient-supplemented perfusate allows four-day ex vivo metabolic preservation of human kidneys. Nat. Commun..

[67] Lin H., Bousnina K., Slagter J.S., Fang Y., Cristoferi I., Garrelds I.M., Danser A.H.J., Reinders M.E.J., Minnee R.C., Hoogduijn M.J. (2025). (Pro)renin, Erythropoietin, Vitamin D and Urodilatin Release From Human Donor Kidneys During Normothermic Machine Perfusion: Predictors of Early Post-Transplant Outcome?. Clin. Transplant..

[68] Leeuwen L.L., Spraakman N.A., Brat A., Huang H., Thorne A.M., Bonham S., van Balkom B.W.M., Ploeg R.J., Kessler B.M., Leuvenink H.G.D. (2021). Proteomic analysis of machine perfusion solution from brain dead donor kidneys reveals that elevated complement, cytoskeleton and lipid metabolism proteins are associated with 1-year outcome. Transpl. Int..

[69] Mulvey J.F., Shaheed S.U., Charles P.D., Snashall C., Lo Faro M.L., Sutton C.W., Jochmans I., Pirenne J., van Kooten C., Leuvenink H.G.D. (2023). Perfusate Proteomes Provide Biological Insight Into Oxygenated Versus Standard Hypothermic Machine Perfusion in Kidney Transplantation. Ann. Surg..

[70] Faucher Q., Alarcan H., Sauvage F.L., Forestier L., Miquelestorena-Standley E., Nadal-Desbarats L., Arnion H., Venhard J.C., Brichart N., Bruyère F. (2022). Perfusate Metabolomics Content and Expression of Tubular Transporters During Human Kidney Graft Preservation by Hypothermic Machine Perfusion. Transplantation.

[71] Werenski H., Stratta R.J., Sharda B., Garner M., Farney A.C., Orlando G., McCracken E., Jay C.L. (2023). Knowing When to Ignore the Numbers: Single-Center Experience Transplanting Deceased Donor Kidneys with Poor Perfusion Parameters. J. Am. Coll. Surg..

[72] Longchamp A., Klauser A., Songeon J., Agius T., Nastasi A., Ruttiman R., Moll S., Meier R.P.H., Buhler L., Corpataux J.M., Lazeyras F. (2020). Ex Vivo Analysis of Kidney Graft Viability Using 31P Magnetic Resonance Imaging Spectroscopy. Transplantation.

[73] Ardehali A., Esmailian F., Deng M., Soltesz E., Hsich E., Naka Y., Mancini D., Camacho M., Zucker M., Leprince P. (2015). Ex-vivo perfusion of donor hearts for human heart transplantation (PROCEED II): a prospective, open-label, multicentre, randomised non-inferiority trial. Lancet.

[74] Hamed A., Tsui S., Huber J., Lin R., Poggio E.C., Ardehali A. (2009). Serum Lactate Is a Highly Sensitive and Specific Predictor of Post Cardiac Transplant Outcomes Using the Organ Care System. J. Heart Lung Transplant..

[75] Graf S., Egle M., Sanz M.N., Segiser A., Clavier A., Arnold M., Gsponer D., Bartkevics M., Kadner A., Siepe M. (2025). Circulating factors, in both donor and ex-situ heart perfusion, correlate with heart recovery in a pig model of DCD. J. Heart Lung Transplant..

[76] McCaig A., Sage A., Keshavjee S., Liu M. (2026). Biomarkers for human donor lung assessment during ex vivo lung perfusion. J. Heart Lung Transplant..

[77] Major T., Ball A.L., Stone J.P., Edge R.J., Lopez-Castejon G., Sjöberg T., Andreasson A., Brough D., Steen S., Fisher A.J., Fildes J.E. (2021). Pro-IL-1β Is an Early Prognostic Indicator of Severe Donor Lung Injury During Ex Vivo Lung Perfusion. Transplantation.

[78] Hashimoto K., Cypel M., Juvet S., Saito T., Zamel R., Machuca T.N., Hsin M., Kim H., Waddell T.K., Liu M., Keshavjee S. (2018). Higher M30 and high mobility group box 1 protein levels in ex vivo lung perfusate are associated with primary graft dysfunction after human lung transplantation. J. Heart Lung Transplant..

[79] Andreasson A.S.I., Karamanou D.M., Gillespie C.S., Özalp F., Butt T., Hill P., Jiwa K., Walden H.R., Green N.J., Borthwick L.A. (2017). Profiling inflammation and tissue injury markers in perfusate and bronchoalveolar lavage fluid during human ex vivo lung perfusion. Eur. J. Cardio. Thorac. Surg..

[80] Kanou T., Nakahira K., Choi A.M., Yeung J.C., Cypel M., Liu M., Keshavjee S. (2021). Cell-free DNA in human ex vivo lung perfusate as a potential biomarker to predict the risk of primary graft dysfunction in lung transplantation. J. Thorac. Cardiovasc. Surg..

[81] Sage A.T., Donahoe L.L., Shamandy A.A., Mousavi S.H., Chao B.T., Zhou X., Valero J., Balachandran S., Ali A., Martinu T. (2023). A machine-learning approach to human ex vivo lung perfusion predicts transplantation outcomes and promotes organ utilization. Nat. Commun..

[82] Sage A.T., Richard-Greenblatt M., Zhong K., Bai X.H., Snow M.B., Babits M., Ali A., Baciu C., Yeung J.C., Liu M. (2021). Prediction of donor related lung injury in clinical lung transplantation using a validated ex vivo lung perfusion inflammation score. J. Heart Lung Transplant..

[83] Yang Z.C., Lu W.Y., Geng Z.Y., Zhao Y., Chen X.M., Zheng T., Wu J.Z., Huang K.J., Yuan H.X., Yang Y. (2025). Identification and validation of biomarkers related to mitochondria during ex vivo lung perfusion for lung transplants based on machine learning algorithm. Gene.

[84] Mesnard B., Ogbemudia E., Bruneau S., Le Bas-Bernardet S., Minault D., Hervouet J., Kervella D., Masset C., Cantarovich D., Rigaud J. (2025). Pancreas Preservation: Hypothermic Oxygenated Perfusion to Improve Graft Reperfusion. Transplantation.

[85] Prudhomme T., Kervella D., Ogbemudia A.E., Gauttier V., Le Bas-Bernardet S., Minault D., Hervouet J., Cantarovich D., Karam G., Renaudin K. (2021). Successful pancreas allotransplantations after hypothermic machine perfusion in a novel diabetic porcine model: a controlled study. Transpl. Int..

[86] Ray S., Parmentier C., Kawamura M., Ganesh S., Nogueira E., Novoa F.C., Hobeika C., Chu T., Kalimuthu S.N., Selzner M., Reichman T.W. (2024). Reanimating Pancreatic Grafts Subjected to Prolonged Cold Ischemic Injury Using Normothermic Ex Vivo Perfusion. Transplant. Direct.

[87] Parmentier C., Ray S., Mazilescu L.I., Kawamura M., Noguchi Y., Nogueira E., Ganesh S., Arulratnam B., Kalimuthu S.N., Selzner M., Reichman T.W. (2023). Normothermic Ex Vivo Machine Perfusion of Discarded Human Pancreas Allografts: A Feasibility Study. Transpl. Int..

[88] Leemkuil M., Lier G., Engelse M.A., Ploeg R.J., de Koning E.J.P., t Hart N.A., Krikke C., Leuvenink H.G.D. (2018). Hypothermic Oxygenated Machine Perfusion of the Human Donor Pancreas. Transplant. Direct.

[89] Prudhomme T., Renaudin K., Lo Faro M.L., Cantarovich D., Kervella D., Minault D., Hervouet J., Le Bas-Bernardet S., Karam G., Blancho G., Branchereau J. (2020). Ex situ hypothermic perfusion of nonhuman primate pancreas: A feasibility study. Artif. Organs.

[90] Yuan F.Z., Shui W., Xing Y.L., Niu Y.Y., Zhang X., Zhang C.R. (2022). Association of Red Cell Index and Adverse Hospitalization Outcomes in Chronic Obstructive Pulmonary Disease Patients with Acute Exacerbation: A Retrospective Cohort Study. Int. J. Chronic Obstr. Pulm. Dis..

[91] Rother T., Horgby C., Schmalkuche K., Burgmann J.M., Nocke F., Jägers J., Schmitz J., Bräsen J.H., Cantore M., Zal F. (2023). Oxygen carriers affect kidney immunogenicity during ex-vivo machine perfusion. Front. Transplant..

[92] Patrono D., Roggio D., Mazzeo A.T., Catalano G., Mazza E., Rizza G., Gambella A., Rigo F., Leone N., Elia V. (2022). Clinical assessment of liver metabolism during hypothermic oxygenated machine perfusion using microdialysis. Artif. Organs.

[93] Ying X., Dong S., Zhao Y., Chen Z., Jiang J., Shi H. (2025). Research Progress on Contrast-Enhanced Ultrasound (CEUS) Assisted Diagnosis and Treatment in Liver-Related Diseases. Int. J. Med. Sci..

[94] Lee Y.H., Kim J.S., Song S.H., Song S.H., Shin H.S., Yang J., Ahn C., Jeong K.H., Hwang H.S. (2022). Impact of donor hypertension on graft survival and function in living and deceased donor kidney transplantation: a nationwide prospective cohort study. J. Hypertens..

[95] Helanterä I., Ibrahim H.N., Lempinen M., Finne P. (2020). Donor Age, Cold Ischemia Time, and Delayed Graft Function. Clin. J. Am. Soc. Nephrol..

[96] Chen Q., Guo J., Han S., Wang T., Xia K., Yu B., Liu Y., Qiu T., Zhou J. (2024). The impact of donor diabetes on recipient postoperative complications, renal function, and survival rate in deceased donor kidney transplantation: a single-center analysis. Ren. Fail..

[97] Markmann J.F., Abouljoud M.S., Ghobrial R.M., Bhati C.S., Pelletier S.J., Lu A.D., Ottmann S., Klair T., Eymard C., Roll G.R. (2022). Impact of Portable Normothermic Blood-Based Machine Perfusion on Outcomes of Liver Transplant: The OCS Liver PROTECT Randomized Clinical Trial. JAMA Surg..

[98] Panayotova G.G., Lunsford K.E., Quillin R.C., Rana A., Agopian V.G., Lee-Riddle G.S., Markovic D., Paterno F., Griesemer A.D., Amin A. (2024). Portable hypothermic oxygenated machine perfusion for organ preservation in liver transplantation: A randomized, open-label, clinical trial. Hepatology.

[99] Hoyer D.P., Paul A., Gallinat A., Molmenti E.P., Reinhardt R., Minor T., Saner F.H., Canbay A., Treckmann J.W., Sotiropoulos G.C., Mathé Z. (2015). Donor information based prediction of early allograft dysfunction and outcome in liver transplantation. Liver Int..

[100] Yang L., Cao H., Sun D., Hou B., Lin L., Shen Z.Y., Song H.L. (2020). Bone marrow mesenchymal stem cells combine with normothermic machine perfusion to improve rat donor liver quality-the important role of hepatic microcirculation in donation after circulatory death. Cell Tissue Res..

[101] Cao H., Wu L., Tian X., Zheng W., Yuan M., Li X., Tian X., Wang Y., Song H., Shen Z. (2021). HO-1/BMMSC perfusion using a normothermic machine perfusion system reduces the acute rejection of DCD liver transplantation by regulating NKT cell co-inhibitory receptors in rats. Stem Cell Res. Ther..

[102] De Stefano N., Navarro-Tableros V., Roggio D., Calleri A., Rigo F., David E., Gambella A., Bassino D., Amoroso A., Patrono D. (2021). Human liver stem cell-derived extracellular vesicles reduce injury in a model of normothermic machine perfusion of rat livers previously exposed to a prolonged warm ischemia. Transpl. Int..

[103] Sun D., Yang L., Zheng W., Cao H., Wu L., Song H. (2021). Protective Effects of Bone Marrow Mesenchymal Stem Cells (BMMSCS) Combined with Normothermic Machine Perfusion on Liver Grafts Donated After Circulatory Death via Reducing the Ferroptosis of Hepatocytes. Med. Sci. Monit..

[104] Thompson E.R., Bates L., Ibrahim I.K., Sewpaul A., Stenberg B., McNeill A., Figueiredo R., Girdlestone T., Wilkins G.C., Wang L. (2021). Novel delivery of cellular therapy to reduce ischemia reperfusion injury in kidney transplantation. Am. J. Transplant..

[105] Del Turco S., Cappello V., Tapeinos C., Moscardini A., Sabatino L., Battaglini M., Melandro F., Torri F., Martinelli C., Babboni S. (2022). Cerium oxide nanoparticles administration during machine perfusion of discarded human livers: A pilot study. Liver Transplant..

[106] Nazzal M., Madsen E.C., Armstrong A., van Nispen J., Murali V., Song E., Voigt M., Madnawat H., Welu A., Manithody C. (2022). Novel NMP split liver model recapitulates human IRI and demonstrates ferroptosis modulators as a new therapeutic strategy. Pediatr. Transplant..

[107] Bonaccorsi-Riani E., Gillooly A.R., Iesari S., Brüggenwirth I.M.A., Ferguson C.M., Komuta M., Xhema D., Daumerie A., Maistriaux L., Leuvenink H. (2022). Delivering siRNA Compounds During HOPE to Modulate Organ Function: A Proof-of-concept Study in a Rat Liver Transplant Model. Transplantation.

[108] Thompson E.R., Sewpaul A., Figuereido R., Bates L., Tingle S.J., Ferdinand J.R., Situmorang G.R., Ladak S.S., Connelly C.M., Hosgood S.A. (2022). MicroRNA antagonist therapy during normothermic machine perfusion of donor kidneys. Am. J. Transplant..

[109] van Leeuwen L.L., Ruigrok M.J.R., Kessler B.M., Leuvenink H.G.D., Olinga P. (2024). Targeted delivery of galunisertib using machine perfusion reduces fibrogenesis in an integrated ex vivo renal transplant and fibrogenesis model. Br. J. Pharmacol..

