# Supplementary material for: The potential biomarkers of organ transplantation in the era of machine perfusion: Current status, challenges, and future directions
Source: iScience. 2026 Oct 28;29(11):117835. doi: 10.1016/j.isci.2026.117835 (PMC13738173; doi:10.1016/j.isci.2026.117835)

iScience, Volume *29*

Supplemental information

The potential biomarkers of organ transplantation in the era of machine perfusion: Current status, challenges, and future directions

Zongwan Yang, Xiaoqing Ying, Zhaojie Guan, Shangxin Dong, Yuanyuan Zhao, Huibo Shi, and Jipin Jiang

The development of machine perfusion (MP) :

As early as the 1850s, Claude Bernard described equipment for liver perfusion [1]. The first recorded perfusion experiment was conducted in 1849 by Carl Eduard Loebell, who perfused an isolated pig kidney to investigate how renal perfusion volume and ureteral pressure influence urine secretion [2]. In 1885, Max von Frey and Max Gruber developed the first closed perfusion system[3]. The first preclinical application of MP occurred in 1922, when a heart-lung apparatus was employed to perfuse an isolated kidney while allowing independent control of temperature, flow, and pressure [4]. The same team subsequently adapted this system to perfuse two kidneys simultaneously [5]. A major milestone came with the Lindbergh perfusion pump, which enabled sterile organ perfusion and marked the beginning of a new era in MP technology[6]. Later, Clarence Dennis pioneered the clinical use of the heart-lung machine, first applied in a 6-year-old girl [7]. Building on these advances, the first successful ex situ MP experiments were achieved in the liver in 1966[8] and in the kidney in 1968[9]. A landmark multicenter randomised controlled trial in 2009 demonstrated the clinical superiority of HMP over SCS in KT[10]. To date, the range of MP equipment includes: HMP, NMP, SNMP and HOPE.The perfusion pump developed by Lindbergh enabled the thyroid to be preserved under sterile conditions at room temperature[11, 12], and this became the prototype for subsequent developments in NMP equipment. The main commercial NMP devices currently available include OrganOx (UK), TransMedics (US), Xvivo (US) and Organ Assit (NLD)[13]. In 2012, NMP first used in human discard livers[14]. A landmark randomized controlled trial has demonstrated the efficacy of NMP in reducing liver

damage[15]. Subsequently, a multicenter randomized controlled trial of portable NMP in liver transplantation yielded promising results[16]. With the development of NMP, long-term perfusion, spanning days to weeks, provides a window for organ modification prior to transplantation[17-19].

1850s, Claude Bernard described equipment for liver perfusion	MARTINS P N, BUCHWALD J E, MERGENTAL H, et al. The role of normothermic machine perfusion in liver transplantation [J]. Int J Surg, 2020, 82s: 52-60.
In 1885, Max von Frey and Max Gruber developed the first closed perfusion system.	BOETTCHER W, MERKLE F, WEITKEMPER H H. History of extracorporeal circulation: the conceptional and developmental period [J]. J Extra Corpor Technol, 2003, 35(3): 172-83.
In 1935, A major milestone came with the Lindbergh perfusion pump	DUTKOWSKI P, DE ROUGEMONT O, CLAVIEN P A. Alexis Carrel: genius, innovator and ideologist [J]. Am J Transplant, 2008, 8(10): 1998-2003.
the first successful liver transplantation in 1966 and in the kidney transplantation in 1968	SOUSA DA SILVA R X, WEBER A, DUTKOWSKI P, et al. Machine perfusion in liver transplantation [J]. Hepatology, 2022, 76(5): 1531-49.<br>BELZER F O, ASHBY B S, GULYASSY P F, et al. Successful seventeen-hour preservation and transplantation of human-cadaver kidney [J]. N Engl J Med, 1968, 278(11): 608-10.
In 2009, first RCT in kidney transplantation	MOERS C, SMITS J M, MAATHUIS M H, et al. Machine perfusion or cold storage in deceased-donor kidney transplantation [J]. N Engl J Med, 2009, 360(1): 7-19.
Foundation of NMP: Lindbergh perfusion pump	LINDBERGH C A. AN APPARATUS FOR THE CULTURE OF WHOLE ORGANS [J]. J Exp Med, 1935, 62(3): 409-31.<br>CARREL A, LINDBERGH C A. THE CULTURE OF WHOLE ORGANS [J]. Science, 1935, 81(2112): 621-3.

The main commercial NMP devices currently available include OrganOx (UK), TransMedics (US), Xvivo (US) and Organ Assit (NLD)	JAYANT K, RECCIA I, VIRDIS F, et al. The Role of Normothermic Perfusion in Liver Transplantation (TRaNsIT Study): A Systematic Review of Preliminary Studies [J]. HPB Surg, 2018, 2018: 6360423.
In 2012, NMP first used in discard human livers	OP DEN DRIES S, KARIMIAN N, SUTTON M E, et al. Ex vivo normothermic machine perfusion and viability testing of discarded human donor livers [J]. Am J Transplant, 2013, 13(5): 1327-35.
In 2018, a landmark randomized controlled trial has demonstrated the efficacy of NMP in reducing liver damage	NASRALLA D, COUSSIOS C C, MERGENTAL H, et al. A randomized trial of normothermic preservation in liver transplantation [J]. Nature, 2018, 557(7703): 50-6.
In 2022, a multicenter randomized controlled trial of portable NMP in liver transplantation yielded promising results	MARKMANN J F, ABOULJOUD M S, GHOBRIAL R M, et al. Impact of Portable Normothermic Blood-Based Machine Perfusion on Outcomes of Liver Transplant: The OCS Liver PROTECT Randomized Clinical Trial [J]. JAMA Surg, 2022, 157(3): 189-98.
NMP, long-term perfusion, spanning days to weeks, provides a window for organ modification prior to transplantation	LAU N S, MCCAUGHAN G, LY M, et al. Long-term machine perfusion of human split livers: a new model for regenerative and translational research [J]. Nat Commun, 2024, 15(1): 9809.<br>LAU N S, LY M, DENNIS C, et al. Long-term ex situ normothermic perfusion of human split livers for more than 1 week [J]. Nat Commun, 2023, 14(1): 4755.<br>LY M, LAU N S, DENNIS C, et al. Long-term ex situ normothermic machine perfusion allows regeneration of human livers with severe bile duct injury [J]. Am J Transplant, 2025, 25(1): 60-71.

[1] MARTINS P N, BUCHWALD J E, MERGENTAL H, et al. The role of normothermic machine perfusion in liver transplantation [J]. Int J Surg, 2020, 82s: 52-60.

[2] BOETTCHER W, MERKLE F, WEITKEMPER H H. History of extracorporeal circulation: the conceptional and developmental period [J]. J Extra Corpor Technol, 2003, 35(3): 172-83.

[3] SZENTGYORGYI L, BUTT S P, KRISHNAMOORTHY B. Evolution of extracorporeal

membrane oxygenation: historical milestones and advanced developments [J]. J Extra Corpor Technol, 2025, 57(4): 243-62.
[4] VERNEY E B, STARLING E H. On secretion by the isolated kidney [J]. J Physiol, 1922, 56(5): 353-8.
[5] EICHHOLTZ F, STARLING E H. The action of inorganic salts on the secretion of the isolated kidney [J]. Proceedings of the Royal Society of London Series B, Containing Papers of a Biological Character, 1925, 98(687): 93-113.
[6] DUTKOWSKI P, DE ROUGEMONT O, CLAVIEN P A. Alexis Carrel: genius, innovator and ideologist [J]. Am J Transplant, 2008, 8(10): 1998-2003.
[7] DENNIS C, SPRENG D S, JR., NELSON G E, et al. Development of a pump-oxygenator to replace the heart and lungs; an apparatus applicable to human patients, and application to one case [J]. Ann Surg, 1951, 134(4): 709-21.
[8] SOUSA DA SILVA R X, WEBER A, DUTKOWSKI P, et al. Machine perfusion in liver transplantation [J]. Hepatology, 2022, 76(5): 1531-49.
[9] BELZER F O, ASHBY B S, GULYASSY P F, et al. Successful seventeen-hour preservation and transplantation of human-cadaver kidney [J]. N Engl J Med, 1968, 278(11): 608-10.
[10] MOERS C, SMITS J M, MAATHUIS M H, et al. Machine perfusion or cold storage in deceased-donor kidney transplantation [J]. N Engl J Med, 2009, 360(1): 7-19.
[11] LINDBERGH C A. AN APPARATUS FOR THE CULTURE OF WHOLE ORGANS [J]. J Exp Med, 1935, 62(3): 409-31.
[12] CARREL A, LINDBERGH C A. THE CULTURE OF WHOLE ORGANS [J]. Science, 1935, 81(2112): 621-3.
[13] JAYANT K, RECCIA I, VIRDIS F, et al. The Role of Normothermic Perfusion in Liver Transplantation (TRaNsIT Study): A Systematic Review of Preliminary Studies [J]. HPB Surg, 2018, 2018: 6360423.
[14] OP DEN DRIES S, KARIMIAN N, SUTTON M E, et al. Ex vivo normothermic machine perfusion and viability testing of discarded human donor livers [J]. Am J Transplant, 2013, 13(5): 1327-35.
[15] NASRALLA D, COUSSIOS C C, MERGENTAL H, et al. A randomized trial of normothermic preservation in liver transplantation [J]. Nature, 2018, 557(7703): 50-6.
[16] MARKMANN J F, ABOULJOUD M S, GHOBRIAL R M, et al. Impact of Portable Normothermic Blood-Based Machine Perfusion on Outcomes of Liver Transplant: The OCS Liver PROTECT Randomized Clinical Trial [J]. JAMA Surg, 2022, 157(3): 189-98.
[17] LAU N S, MCCAUGHAN G, LY M, et al. Long-term machine perfusion of human split livers: a new model for regenerative and translational research [J]. Nat Commun, 2024, 15(1): 9809.
[18] LAU N S, LY M, DENNIS C, et al. Long-term ex situ normothermic perfusion of human split livers for more than 1 week [J]. Nat Commun, 2023, 14(1): 4755.
[19] LY M, LAU N S, DENNIS C, et al. Long-term ex situ normothermic machine perfusion allows regeneration of human livers with severe bile duct injury [J]. Am J Transplant, 2025, 25(1): 60-71.

[20] SIMCHON S, CHIEN S. A new method for kidney perfusion in situ: application to dynamics of autoregulation [J]. Am J Physiol, 1982, 242(1): F86-94.
[21] LIM M W. The history of extracorporeal oxygenators [J]. Anaesthesia, 2006, 61(10): 984-95.

The references for the literature cited in Figure 2 regarding developments in pumps and oxygenation devices used in MP are as follows:

Gravity column	BOETTCHER W, MERKLE F, WEITKEMPER H H. History of extracorporeal circulation: the conceptional and developmental period [J]. J Extra Corpor Technol, 2003, 35(3): 172-83.
Pulsatile syringe	SIMCHON S, CHIEN S. A new method for kidney perfusion in situ: application to dynamics of autoregulation [J]. Am J Physiol, 1982, 242(1): F86-94.
Balloon pulse pump	von Schröder, W. (1882). *Ueber die Bildungsstätte des Harnstoffs*. Archiv für experimentelle Pathologie und Pharmakologie, 15, 364-400.
Electric syringe pump and Electric roller pump	BOETTCHER W, MERKLE F, WEITKEMPER H H. History of extracorporeal circulation: the conceptional and developmental period [J]. J Extra Corpor Technol, 2003, 35(3): 172-83.
Modern roller pump	ESHMUMINOV D, LEONI F, SCHNEIDER M A, et al. Perfusion settings and additives in liver normothermic machine perfusion with red blood cells as oxygen carrier. A systematic review of human and porcine perfusion protocols [J]. Transpl Int, 2018.<br>DONDOSSOLA D, DE FALCO S, KERSIK A, et al. Procurement and ex-situ perfusion of isolated slaughterhouse-derived livers as a model of donors after circulatory death [J]. Altex, 2019.
Stirring aeration	LIM M W. The history of extracorporeal oxygenators [J]. Anaesthesia, 2006, 61(10): 984-95.
Bubble oxygenator	LIM M W. The history of extracorporeal oxygenators [J]. Anaesthesia, 2006, 61(10): 984-95.<br>SZENTGYORGYI L, BUTT S P,

	KRISHNAMOORTHY B. Evolution of extracorporeal membrane oxygenation: historical milestones and advanced developments [J]. J Extra Corpor Technol, 2025, 57(4): 243-62.
Film oxygenator	LIM M W. The history of extracorporeal oxygenators [J]. Anaesthesia, 2006, 61(10): 984-95.<br>SZENTGYORGYI L, BUTT S P, KRISHNAMOORTHY B. Evolution of extracorporeal membrane oxygenation: historical milestones and advanced developments [J]. J Extra Corpor Technol, 2025, 57(4): 243-62.
Closed system bubble oxygenator	BOETTCHER W, MERKLE F, WEITKEMPER H H. History of extracorporeal circulation: the conceptional and developmental period [J]. J Extra Corpor Technol, 2003, 35(3): 172-83.
Rotary disc oxygenator	LIM M W. The history of extracorporeal oxygenators [J]. Anaesthesia, 2006, 61(10): 984-95.<br>KRISHNAMOORTHY B. Evolution of extracorporeal membrane oxygenation: historical milestones and advanced developments [J]. J Extra Corpor Technol, 2025, 57(4): 243-62.
Glass bead oxygenator	KRISHNAMOORTHY B. Evolution of extracorporeal membrane oxygenation: historical milestones and advanced developments [J]. J Extra Corpor Technol, 2025, 57(4): 243-62.
Spiral tube film oxygenator	BOETTCHER W, MERKLE F, WEITKEMPER H H. History of extracorporeal circulation: the conceptional and developmental period [J]. J Extra Corpor Technol, 2003, 35(3): 172-83.

Conceptual sketch of the classification of biomarkers in Figure 2:

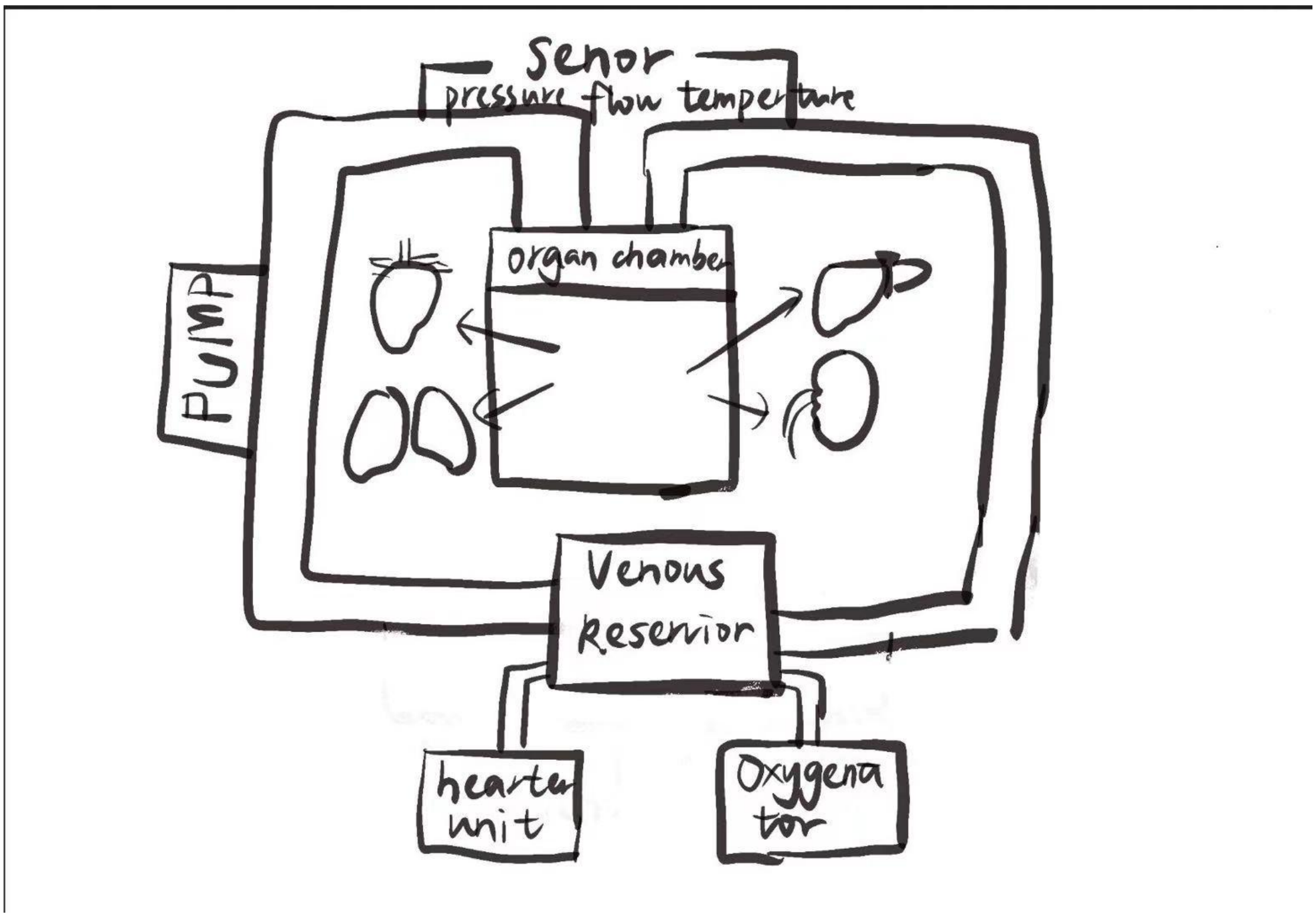
Senor
pressure flow temperature
PUMP
organ chamber
Venous Reservior
hearter unit
Oxygenator

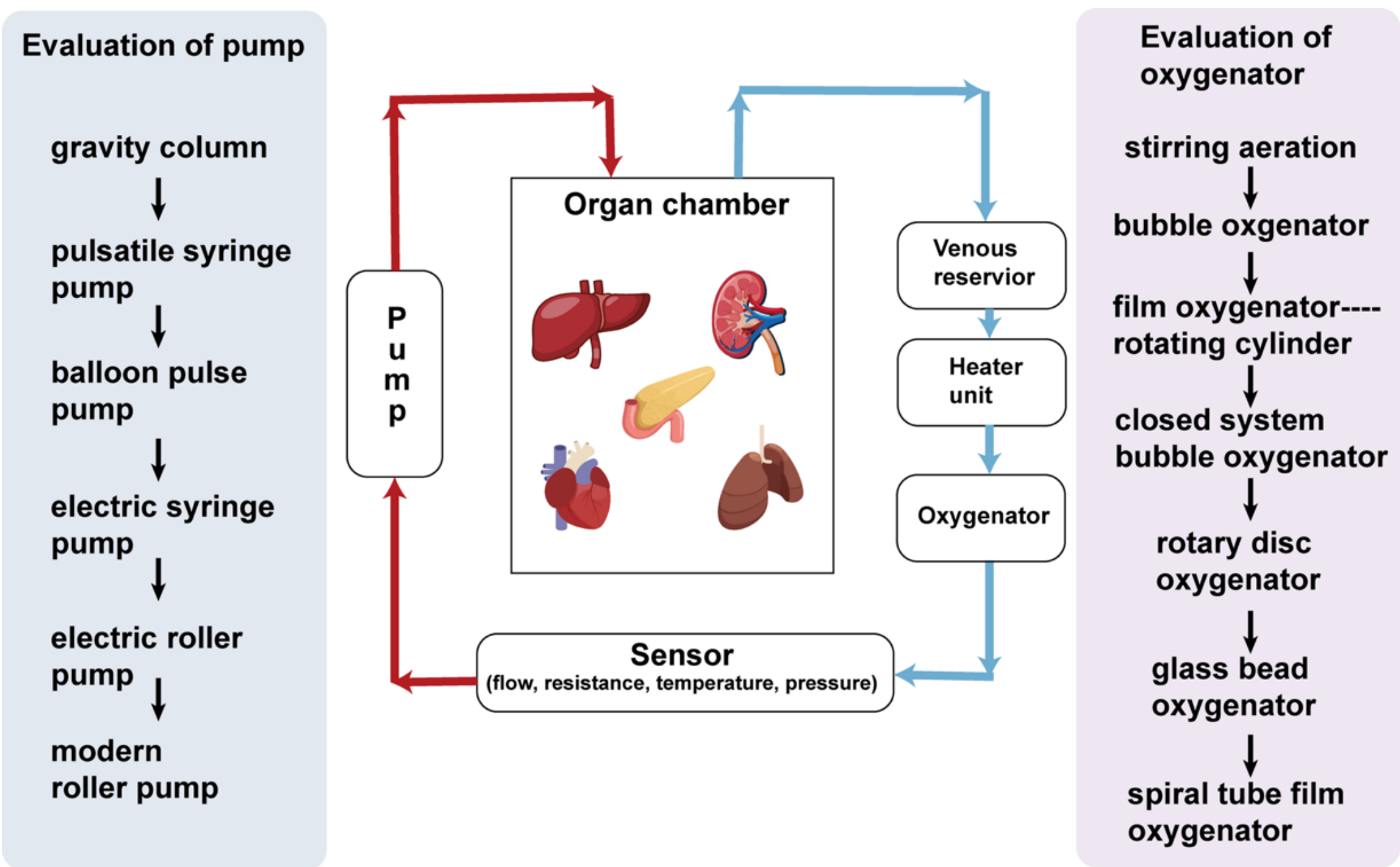
Evaluation of pump
gravity column
pulsatile syringe pump
balloon pulse pump
electric syringe pump
electric roller pump
modern roller pump
Pump
Organ chamber
Venous reservior
Heater unit
Oxygenator
Sensor
(flow, resistance, temperature, pressure)
Evaluation of oxygenator
stirring aeration
bubble oxgenator
film oxygenator----rotating cylinder
closed system bubble oxygenator
rotary disc oxygenator
glass bead oxygenator
spiral tube film oxygenator

Conceptual sketch of the classification of biomarkers in Figure 3:

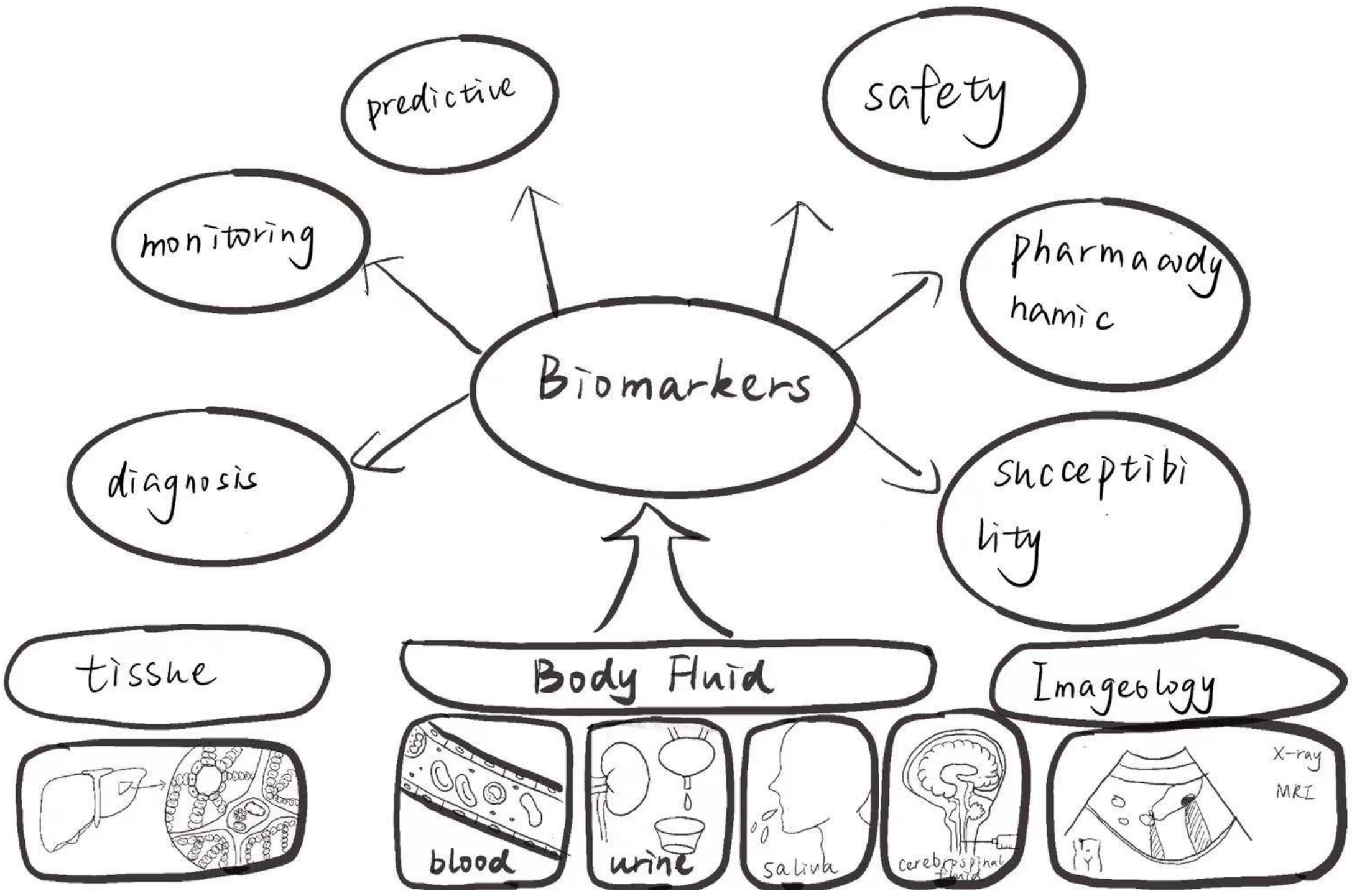

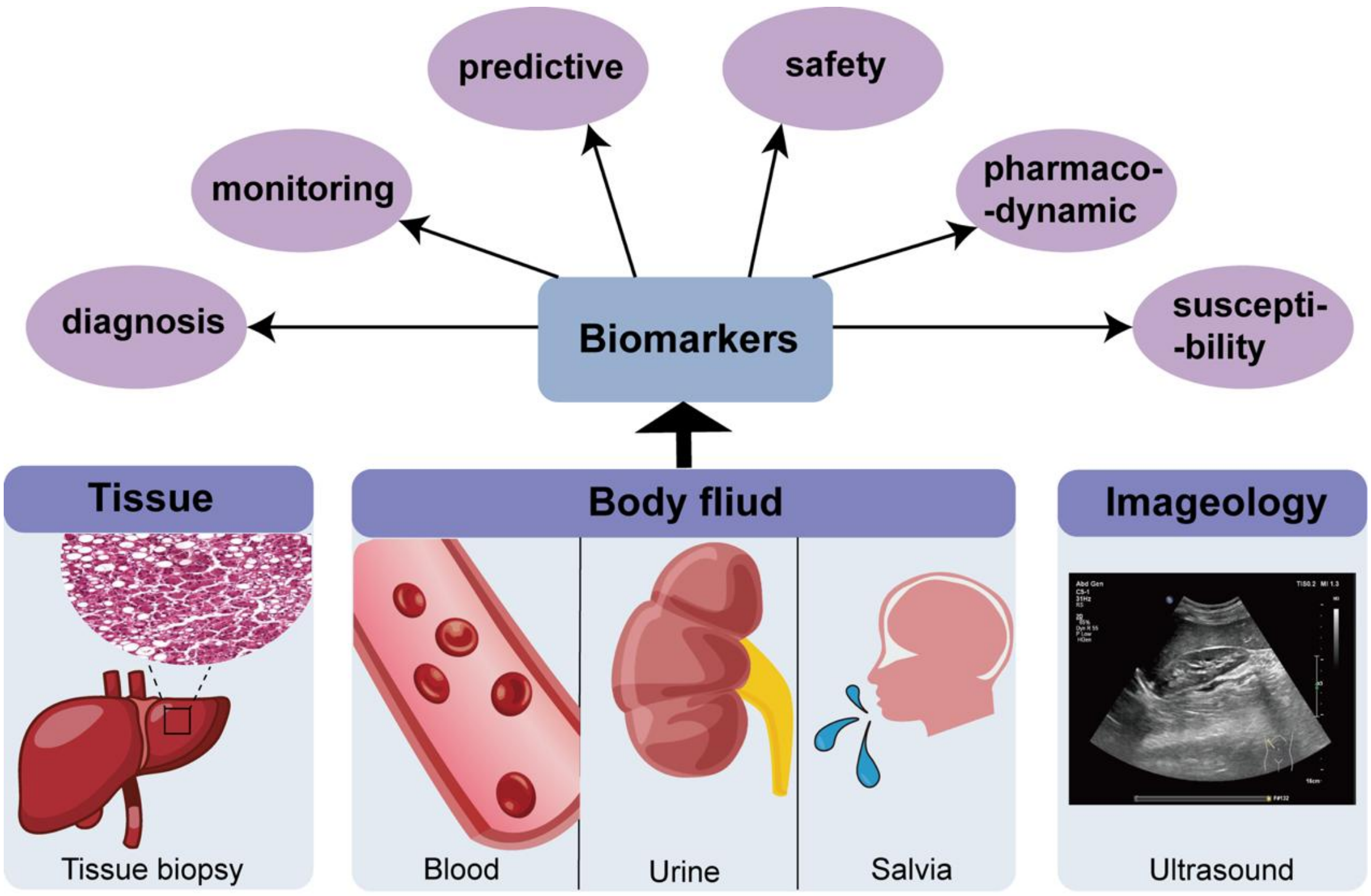

The content in Figure 4 has been revised to reflect changes made to the “Challenge and Future Directions” sections; therefore, the content in the figure has been updated accordingly.

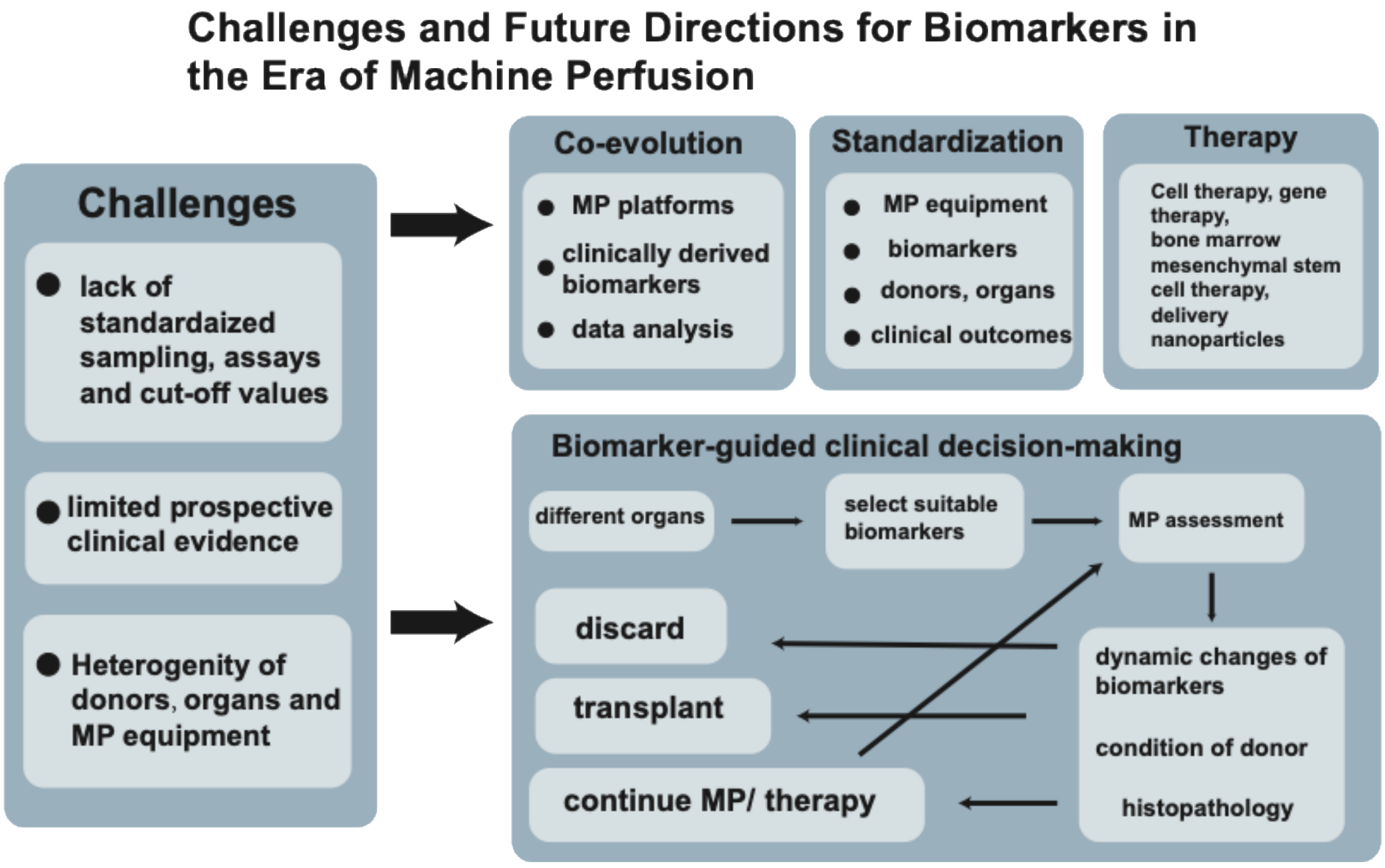

Supplement: Supplementary file 1 [file mmc1.pdf]
